# Is spaceflight-induced immune dysfunction linked to systemic changes in metabolism?

**DOI:** 10.1371/journal.pone.0174174

**Published:** 2017-05-24

**Authors:** Michael J. Pecaut, Xiao Wen Mao, Denise L. Bellinger, Karen R. Jonscher, Louis S. Stodieck, Virginia L. Ferguson, Ted A. Bateman, Robert P. Mohney, Daila S. Gridley

**Affiliations:** 1 Department of Basic Sciences, Division of Radiation Research, Loma Linda University School of Medicine, Loma Linda, CA, United States of America; 2 Department of Anatomy, Loma Linda University, Loma Linda, CA, United States of America; 3 Department of Anesthesiology, School of Medicine, University of Colorado Anschutz Medical Campus, Denver, CO, United States of America; 4 BioServe Space Technologies, University of Colorado at Boulder, Boulder, CO, United States of America; 5 Department of Bioengineering, University of North Carolina at Chapel Hill, Chapel Hill, NC, United States of America; 6 Metabolon, Inc., Morrisville, NC, United States of America; Georgetown University, UNITED STATES

## Abstract

The Space Shuttle *Atlantis* launched on its final mission (STS-135) on July 8, 2011. After just under 13 days, the shuttle landed safely at Kennedy Space Center (KSC) for the last time. Female C57BL/6J mice flew as part of the Commercial Biomedical Testing Module-3 (CBTM-3) payload. Ground controls were maintained at the KSC facility. Subsets of these mice were made available to investigators as part of NASA’s Bio-specimen Sharing Program (BSP). Our group characterized cell phenotype distributions and phagocytic function in the spleen, catecholamine and corticosterone levels in the adrenal glands, and transcriptomics/metabolomics in the liver. Despite decreases in most splenic leukocyte subsets, there were increases in reactive oxygen species (ROS)-related activity. Although there were increases noted in corticosterone levels in both the adrenals and liver, there were no significant changes in catecholamine levels. Furthermore, functional analysis of gene expression and metabolomic profiles suggest that the functional changes are not due to oxidative or psychological stress. Despite changes in gene expression patterns indicative of increases in phagocytic activity (e.g. endocytosis and formation of peroxisomes), there was no corresponding increase in genes related to ROS metabolism. In contrast, there were increases in expression profiles related to fatty acid oxidation with decreases in glycolysis-related profiles. Given the clear link between immune function and metabolism in many ground-based diseases, we propose a similar link may be involved in spaceflight-induced decrements in immune and metabolic function.

## Introduction

The final US Space Shuttle mission, STS-135, launched on July 8, 2011. After 12 days, 18 hours and 29 minutes, Space Shuttle *Atlantis* landed safely at Kennedy Space Center (KSC) for the last time. One of the many experiments flown on this historic flight to the International Space Station (ISS) was BioServe Space Technology’s Commercial Biomedical Testing Module-3 (CBTM-3). Sponsored by Amgen, Inc., the last animal flight of the shuttle era was intended to test the impact of their proprietary pharmaceutical agent on spaceflight-induced musculoskeletal atrophy. Participants in the NASA Biospecimen Sharing Program (BSP) were given the opportunity to analyze a subset of the placebo-treated mice (n = 5–10, depending on the assay and tissue) as long as it did not interfere with the primary science. Our research team at Loma Linda University (LLU) has participated in all three Amgen/CBTM flights (STS-108, -118, and -135) [1–6], giving us the unique opportunity to both repeat and expand on previous results.

There are at least three, currently unavoidable components of the spaceflight environment that directly impact immune function. These include changes in the inertial environment (e.g. launch and landing loads, microgravity), low-dose/low-dose-rate radiation (e.g. solar particle events, galactic cosmic rays, and the Van Allen belts), and physiological/psychological stress (e.g. unloading, work-related stress). Ground-based studies have shown that each of these environmental factors alone can have an impact on immune function.

Previously, we and others have shown that the spaceflight environment can have a dramatic influence on immunity. Virtually all immune populations are reduced after spaceflight [2, 4]. Studies in both animal models and humans have shown that the spaceflight environment can influence total body, thymus and spleen mass [7–18], lymphocyte population distributions [18–25] and circulating corticosterone levels [16, 17, 26–33].

Interestingly, spaceflight is also known to alter energy/lipid metabolism [34, 35] and ground-based studies suggest that the stress marker, corticosterone/cortisol, plays a significant role [36]. Corticosterone promotes fat breakdown in adipose and muscle tissues to provide glycerol to the liver for gluconeogenesis [36]. As we demonstrate, metabolomic analysis of the liver indicates that glycerol was more abundant in flight mice relative to ground controls. Furthermore, data from the liver, skin and adrenal gland suggest that spaceflight caused a significant increase in corticosterone levels system-wide.

These seemingly unrelated lines of investigation are important because there is a growing body of work describing the interaction between innate immunity and lipid metabolism (S1 Fig). Chronic inflammation is now accepted as a critical component in many pathologies and chronic diseases. Macrophages are not only among the first responders in host resistance to infection, but also have an underappreciated role in host health when metabolic changes occur [37]. For example, in obese people, increased numbers of liver and adipose tissue macrophages correlate with the development of metabolic syndrome [38].

In the current study we describe a more systemic response to microgravity by combining traditional measures of innate immune function (population distributions, oxidative burst capacity, phagocytosis) and stress responses (adrenal catecholamine and corticosterone levels) with ‘omics techniques (metabolomics and transcriptomics). We believe that, for the first time, we can begin to show the links between spaceflight-dependent immune dysfunction and changes in metabolism.

## Methods

### Mice and treatment

On July 8, 2011, age- and weight-matched, 11 week old, female C57BL/6J mice (n = 15/group) were placed into three Animal Enclosure Modules (AEMs, 2 groups of 5 per AEM separated by a wire mesh) and flown on the Space Shuttle Atlantis (STS-135) for ~13 days. There were two sets of ground controls housed at the Space Life Science Laboratory (SLSL) at KSC: Ground AEM controls (n = 10) and Vivarium controls (n = 8). Due to the nature of the NASA Biospecimen Sharing Program (BSP), we did not receive tissues from all mice for all assays. Sample sizes for individual assays are indicated where appropriate.

Ground AEM control mice were placed into the same hardware used in flight and environmental parameters such as temperature and CO_2_ levels were matched as closely as possible based on 48-hour delayed telemetry data. Conditions were controlled for temperature, humidity, and a 12:12 hr light:dark cycle; food (food bars developed by NASA Ames) and water was provided *ad libitum*. Vivarium control mice, housed in standard shoebox cages and fed standard rodent chow, were used to normalize cell counts, thus minimizing day-to-day variability.

Mice were euthanized and dissected within 3–5 hours of landing. As part of the primary science, all mice underwent dual energy X-ray absorptiometry (DEXA) densitometry (Piximus, Inc., Fitchburg, WI) immediately prior to anesthesia and euthanization. Mice were anesthetized with 3–5% isoflurane and euthanized with 100% CO_2_ and exsanguination via cardiac puncture. Samples were shipped to either LLU or University of Colorado Anschutz Medical Campus and stored appropriately prior to use. These studies were approved by the NASA Ames and University of Colorado at Boulder Institutional Animal Care and Use Committees (IACUCs).

### Tissue handling

Each spleen was cut in half at the time of dissection, placed into complete Roswell Park Memorial Institute-1640 (RPMI-1640) medium with 20% bovine calf serum and shipped on ice via overnight courier to LLU for further processing. *Note*: *There was a problem with the courier shipping fresh tissues from the ground control mice from KSC to LLU*. *As a result*, *“fresh” ground control splenocyte samples were processed and assayed at LLU 48 hours after tissue collection*. *In contrast*, *“fresh” flight samples were processed and assayed roughly 24 hours post-tissue collection*. *This must be considered in interpreting all assays involving “fresh” splenocytes presented below*. *Only the assays that involved fresh splenic tissue were affected by this shipping error*. *These include flow cytometry*, *oxidative burst*, *background ROS*, *and phagocytosis assays*.

Adrenals were snap frozen in liquid nitrogen at the time of dissection and stored at -80°C until analysis. Liver lobes were extracted and dissected at the time of dissection. A portion of the sample was prepared in 4% paraformaldehyde. The rest of the liver was snap frozen in liquid nitrogen and stored at -80°C until analysis. *Note*: *none of the liver or adrenal tissues were impacted by the shipping error*.

### Splenocyte viability

Spleen cell concentration was adjusted to 2 x 10^6^ cells/ml and mixed with 0.4% trypan blue solution. The cell suspension was loaded onto CHT4 counting chamber slides and viability was assessed via the Cellometer Auto T4 (Nexcelcom Bioscience LLC, Lawrence, MA).

### Splenic leukocyte quantification

Splenocyte single-cell suspensions were diluted in RPMI-1640 medium and evaluated using the ABC Vet Hematology Analyzer (Heska Corp., Waukesha, WI) after lysis of erythrocytes by incubation in 2 ml of ammonium-chloride-potassium solution for 4 min at 4°C.

Immunophenotyping of lymphocyte subsets was performed on spleen using a FACSCalibur^TM^ flow cytometer (Becton Dickinson, Inc., San Jose, CA). Monoclonal antibodies (MAb; BD Pharmingen, Inc., San Diego, CA) were labeled with fluorescein isothiocyanate (FITC), R-phycoerythrin (PE), allophycocyanin (APC), or peridinin chlorophyll protein (PerCP). MAb were directed against the following markers: CD3 (G4.18)–mature T cells; CD4 (OX-35)–T helper (Th) cells; CD8 (OX-8)–T cytotoxic (Tc) cells; CD45 (OX-1)–leukocytes; CD45R (HIS24)–B cells. We gated on the mononuclear population on a CD45 vs side scatter dot plot. At least 5,000 gated events/tube were evaluated using CellQuest^TM^ software version 3.1 (Becton Dickinson).

All cell count numbers were normalized to Vivarium controls and combined for the final analysis using the following equation: Final Data Point = [Measured Data Point] / [Average of Daily Vivarium Control] * [Average of All Controls for All Experiments].

### Splenocyte oxidative burst and background reactive oxygen species (ROS)–plate reader

Spleen white blood cells (WBCs) were treated with zymosan A (for burst activity; Sigma Chemical Co., St. Louis, MO) or medium (for background activity), and 2',7'-dichlorodihydrofluorescein diacetate (DCFH-DA; Molecular Probes, Inc., Eugene, OR), a membrane-permeable diacetate derivative of DCFH. Upon entering cells the diacetate is cleaved enzymatically. Both the DCFH-DA and DCFH are non-fluorescent fluorescein analogues. They are oxidized to highly fluorescent 2',7'-dichlorofluorescein (DCF) by H_2_O_2_ and hydroperoxides. The fluorescence intensity was measured using a fluorometer at 485 nm excitation and 530 emission.

### Splenocyte oxidative burst and phagocytosis–flow cytometry

Fixed *Staphylococcus aureus* (Trade name Pansorbin, Calbiochem, San Diego, CA) was stained with propidium iodide (PI) and used to quantify phagocytosis. DCFH-DA was used to quantify oxidative burst. Spleen cells were transferred into 5 ml tubes. DCFH-DA and PI-labeled *S*. *aureus* were added to the cells and allowed to incubate. Fluorescence was detected using a 4-color FACSCalibur^TM^ flow cytometer. Analysis of 5,000 events/tube was performed using CellQuest^TM^ software (v3.1).

### Adrenal tissue homogenate

One adrenal gland from each mouse was weighed and homogenized using a Powergen homogenizer (Fischer Scientific, Tustin, CA) with 500 μl of phosphate-buffered saline (PBS), the other adrenal was homogenized with 500 μl of 0.1M HClO_4_. The samples were spun at 12,000 x g for 15 min at 4°C. The supernatant was aliquotted and frozen at -80°C until assayed. The supernatants were used to assess adrenocorticotropic hormone (ACTH) receptor. Samples homogenized in HClO_4_ were used to assess corticosterone, protein, and catecholamines.

### Total protein concentration in adrenals

Total protein concentration was quantified in the adrenals using a Pierce bicinchoninic acid (BCA) Protein Assay kit (Thermo Fisher Scientific, Tustin, CA). Samples were run with protein standards ranging from 500 μg/ml to 7.8 μg/ml diluted with 0.1M HClO_4_.

### Corticosterone via enzyme-linked immunosorbent assay (ELISA)

The adrenal glands from each mouse were homogenized. Supernatants were collected after centrifugation and stored at -80°C. Aliquots of frozen adrenal tissue supernatant were thawed, diluted 1:10 in assay buffer, and corticosterone was measured using an ELISA kit for mouse corticosterone from AssayPro (St. Charles, MO), according to manufacturer’s instructions. Plates containing duplicate samples and standards were read on a microplate reader set at 450 nm. Corticosterone levels in adrenal supernatants were calculated from the standard curve and expressed as means ± SEM in ng/mg protein. Cross reactivity for other steroids, such as aldosterone or progesterone, was <2% or lower. Minimum detection of corticosterone by the kits was 0.156 ng/ml, and intra-assay and inter-assay variabilities were 5.0% and 7.1%, respectively.

### ACTH receptor via ELISA

ACTH receptors and phosphorylated ACTH receptors were assayed in adrenal gland homogenates using ELISA kits from MyBiosource (San Diego, CA) as per the manufacturer’s instructions. Samples were run in duplicate along with known standards in 96-well plates at room temperature. Plates were read on a plate reader set at 450 nm immediately after adding the stop solution. Data were expressed as means ± SEM in pg/mg protein. The sensitivity of the kit was 2.0 pg/ml. The detection range of the kit was 15.6–500.0 pg/ml. Intra-assay coefficient of variation (CV) and inter-assay CV were less than 15%.

### Catecholamines via high-performance liquid chromatography (HPLC)

Frozen aliquots of supernatant from adrenal tissue were thawed and diluted 1:5 in 0.1M HClO_4_. Epinephrine, norepinephrine and dopamine were measured by HPLC using an ESA 582 isocratic pump, an ESA 542 autosampler, a Waters Atlantis T3 150 mm X 4.6 mm C18 reverse phase column, and an ESA Coulochem III detector with a 5011a cell set at +250 mV and -200 mV. The mobile phase was a 100 μM citric acid/phosphate buffer with 20% methanol pH 3.5. The assay volume was 50 μl. Values were expressed as a mean ± SEM in ng/mg protein.

### Metabolomics

Frozen liver samples were shipped to Metabolon, Inc. (Durham, NC) for analysis. Methanol extraction of small molecules was carried out, as described previously [39, 40], using 100 mg of liver tissue per sample. In addition, a small volume of each sample was utilized to create a pooled liver sample that was prepared in parallel and included as a technical replicate to assess variability and sensitivity in measurements, as described [39, 41]. The resulting extract was divided into four fractions for non-targeted metabolomics analysis [39, 40, 42]: one for analysis by ultra-high performance liquid chromatography-tandem mass spectrometry (UPLC-MS/MS; positive mode), one for analysis by UPLC-MS/MS (negative mode), one for analysis by gas chromatography–mass spectrometry (GC-MS), and one sample was reserved for backup.

The UPLC-MS/MS platform utilized a Waters Acquity UPLC with Waters UPLC BEH C18-2.1×100 mm, 1.7 μm columns and a ThermoFisher LTQ mass spectrometer, which included an electrospray ionization source and a linear ion-trap mass analyzer. The instrumentation was set to monitor for positive ions in acidic extracts or negative ions in basic extracts through independent injections. The instrument was set to scan 80–1000 m/z (molecular weight) and alternated between MS and MS/MS scans. Samples destined for analysis by GC-MS were dried under vacuum desiccation for a minimum of 18 hours prior to being derivatized using bis(trimethylsilyl)trifluoroacetamide. Derivatized samples were separated on a 5% diphenyl / 95% dimethyl polysiloxane fused silica column (20 m x 0.18 mm ID; 0.18 μm film thickness) with helium as carrier gas and a temperature ramp from 60° to 340°C in a 17.5 min period. A Thermo-Finnigan Trace DSQ fast-scanning single-quadrupole MS using electron impact ionization (EI) and operated at unit mass resolving power was utilized, and the scan range was from 50–750 m/z.

Metabolites were identified by automated comparison of the ion features in the experimental samples to a reference library of chemical standard entries that included retention time, molecular weight (m/z), preferred adducts, and in-source fragments as well as associated MS spectra, and curated by visual inspection for quality control using software developed at Metabolon [43]. Identification of known chemical entities was based on comparison to metabolomic library entries of more than 4,000 purified standards. Raw area counts for each metabolite were re-scaled to set the medians to 1.0 for metabolites identified. Missing values, assumed to be below the limit of detection of the platform used, were imputed with the observed minimum after normalization. An estimate of the false discovery rate among metabolites identified as significantly different (p<0.05) between space flight livers and AEM controls was calculated to be 22% when applying the q-value method of Storey and Tibshirani [44].

### RNA isolation

RNA isolation was performed using the RNeasy Mini Kit (Qiagen, Inc. Valencia, CA) as per the manufacturer’s instructions. A maximum of 30-mg frozen liver/mouse tissue was used.

### Target preparation/processing for GeneChip analysis

After isolation, RNA was frozen at -80°C and shipped on dry ice to the University of California, Irvine (UCI) Genomics High-Throughput Facility (GHTF) for further analysis. The following protocol was provided by the UCI GHTF.

Isolated total RNA samples were processed as recommended by Affymetrix, Inc. (Affymetrix GeneChip Whole Transcript Sense Target Labeling Assay Manual, Affymetrix, Inc., Santa Clara, CA). In brief, total RNA was initially isolated as described above and then put through an RNeasy spin column (Qiagen, Chatsworth, CA) for further clean up. Eluted total RNAs were quantified by NanoDrop (ThermoScientific, Wilmington, DE) and the concentrations of sample aliquots were adjusted to 100 ng/μl. Total RNA samples were assessed for quality prior to performing target preparation/processing steps by loading approximately 25–250 ng of each sample onto a RNA 6000 Nano LabChip, which was evaluated on an Agilent Biolanalyzer 2100 (Agilent Technologies, Palo Alto, CA).

The Ambion whole transcript (WT) expression kit (Life Technologies, Carlsbad, CA) was used to prepare RNA samples for whole transcriptome microarray analysis. Briefly, random hexamers that were tagged with a T7 promoter were used in first strand synthesis of cDNA. Then, using the T7 promoter, second strand synthesis was performed and the double-stranded cDNA was subsequently used as a template in an *in vitro* transcription reaction to generate many copies of antisense cRNA. Ten μg of antisense cRNA was input into a second cycle cDNA reaction using reverse transcriptase and random hexamers to produce single-stranded DNA in the sense orientation. The single-stranded DNA was fragmented to an average length of 70 bases and then labeled using a recombinant terminal deoxynucleotidyl transferase (TdT) and an Affymetrix proprietary DNA labeling reagent that was covalently linked to biotin. Two μg of the labeled, fragmented single-stranded cDNA was hybridized at 45°C with rotation for 17 hours (Affymetrix GeneChip Hybridization Oven 640) to probe sets present on an Affymetrix GeneChip 1.0ST array. The GeneChip arrays were washed and then stained with streptavidin-phycoerythrin on an Affymetrix Fluidics Station 450 (Fluidics protocol FS450_007). Arrays were scanned using GeneChip Scanner 3000 7G and Command Console Software v. 3.2.3 to produce.CEL intensity files.

The probe cell intensity files (*.CEL) were analyzed in Affymetrix Expression Console software v1.1.1 using the Probe Logarithmic Intensity Error (PLIER) algorithm to generate probe level summarization files (*.CHP). The settings used were algorithm-PLIER v2.0; quantification scale-Linear; quantification type-signal and detection P value; background-perfect match GC composition-based background correction (PM-GCBG); normalization method-sketch-quantile.

Gene expression data were then processed via CARMAweb (Comprehensive R based Microarray Analysis web service) using the significance analysis of microarrays (SAM) method to get fold changes for determining differential expression and p values. The Benjamini-Hochberg method was used to identify false positives. CARMAweb is a free, online tool hosted by Medical University Innsbruck [45].

### Histology and immunohistochemistry

PFA fixed, 5 μm tissue sections were processed for hematoxylin-and-eosin (H&E) staining and immunofluorescence microscopy as described previously [46]. Additional histology sections were processed for Periodic Schiff Stain (PAS) and Picrosirius Red stain. Immunoreactivity was visualized using secondary antibodies conjugated with Alexafluor 488 or Alexafluor 594 at dilutions of 1:500 and 1:250, respectively. Nuclei were stained with 4‘,6-diamidino-2-phenylindole (Sigma). Immunofluorescence images were captured on a Nikon Diaphot fluorescence microscope (Nikon Corporation, Tokyo, Japan) and digitally deconvolved using the No Neighbors algorithm (Slidebook, Denver, CO) as described previously [46]. Histologic images were captured on an Olympus BX51 microscope equipped with a four megapixel Macrofire digital camera (Optronics, Goleta, CA) using the PictureFrame Application 2.3 (Optronics). Cross-polarized light was also used to enhance visualization of Picrosiruis Red stained images as previously described [47]. All images were cropped and assembled using Photoshop CS2 (Adobe Systems, Inc., Mountain View, CA).

### Statistics

Functional analysis of gene expression data provided by the UCI GHTF was performed at LLU using Ingenuity Pathway Analysis (IPA) software (Qiagen, Inc., Redwood City, CA). A z-score of < -2.0 or > 2.0 was considered significant. A P value of overlap < 0.05, indicates that the genes in our data set significantly overlap with the genes known to be involved with the function. Predicted effects are based on the literature and the measured effect of spaceflight. In some cases, the down-regulation of a specific gene may lead to an increase in functional activity and *vice versa*. The z-score represents the combined predicted response of all genes known to be explicitly involved in a particular function. All other data were analyzed using Sigmaplot 12.0 (Systat Software, Inc., San Jose, CA) with an unpaired T-test; *P<*0.05 and *P<*0.1 indicating significance and trending, respectively.

## Results

### Total body mass decreased after spaceflight but not significantly

There were no significant differences in total body mass at the time of sacrifice (AEM 19.5 ± 0.5 g, Flight 18.4 ± 0.5 g). Sample size: n = 8 for AEM ground controls, n = 7 for Flight. However, when calculated as a difference from preflight, baseline mass, we previously reported that spaceflight mice lost significantly more mass compared to AEM controls [35].

### Decrease in splenocyte viability

There was a significant decrease in viability of splenocytes noted in the ground control mice (*P<*0.001); 77% of splenocytes were viable in flight mice while only 68% were viable in ground controls. The difference in viability was likely due to a problem with shipping fresh tissues from the ground control mice from KSC to LLU (as described above). This difference in viability, although relatively small, must be considered in interpreting all assays involving “fresh” splenocytes presented below. Sample size: n = 8 for AEM ground controls, n = 5 for Flight.

### Spaceflight decreased major splenic leukocyte subset counts

There were significant flight-induced decreases in the total number of all major leukocyte populations (*P<*0.001, S2 Fig). In terms of proportions, spaceflight caused a shift toward lymphocytes (*P<*0.001) away from granulocytes (*P<*0.001), with no significant impact on monocyte/macrophage percentages.

### Spaceflight decreased splenic lymphocyte subset counts

Spaceflight caused decreases in the total count of all measured lymphocyte subsets (S3 Fig) including T (*P<*0.001), Th (*P<*0.001), Tc (*P<*0.005), B (*P<*0.001) and NK (*P<*0.05) cells. In contrast, with the exception of an increase in Tc cells percentages (*P<*0.05), there was no significant impact of spaceflight on the proportions of any lymphocytes. The proportional increase in Tc cells led to a significant, flight-induced decrease in the CD4/CD8 ratio (*P<*0.01), i.e., 1.6 for flight mice versus 2.0 for AEM ground controls.

### Spaceflight increased splenic background ROS & oxidative burst but decreased phagocytosis

**Fig 1:**
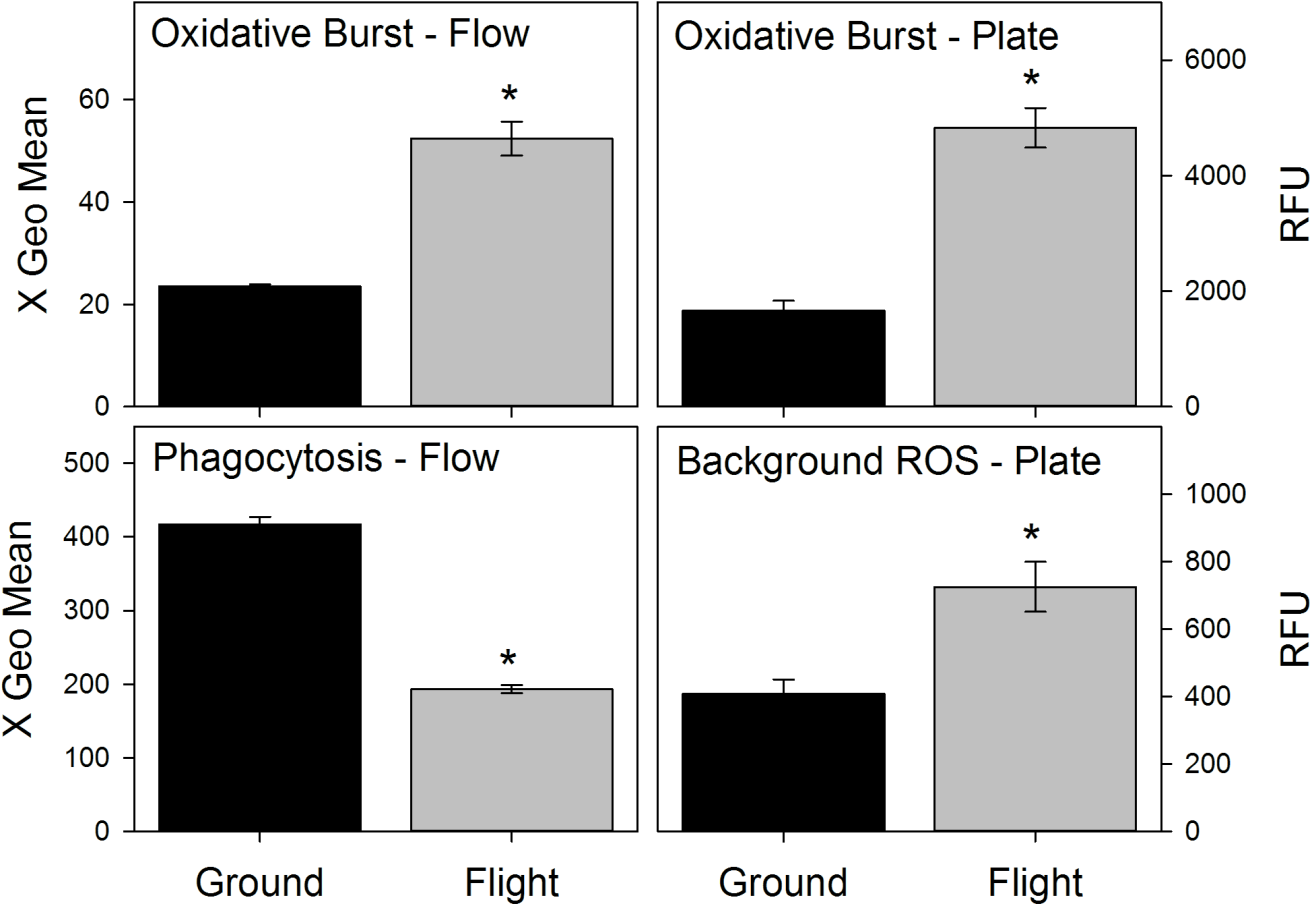
Effects of spaceflight on splenocyte phagocytic function. Flow = Flow cytometry based assay. Plate = 96 well/fluorescence-based assay. Values represent means ± SEM. N = 8 for Ground controls, 5 for Flight. *P<0.001.

As indicated in Fig 1, we characterized the capacity of cells present in the spleen to generate an oxidative burst using both flow cytometry and a plate-based fluorescent assay. In both cases, we found significant spaceflight-induced increases (*P<*0.001). In contrast, there were decreases in phagocytic capacity in flight mice (*P<*0.001). Lastly, spaceflight increased the levels of unstimulated, background ROS (*P<*0.001).

### Spaceflight increased adrenal corticosterone, decreased ACTH receptors, and did not impact catecholamine levels

**Fig 2:**
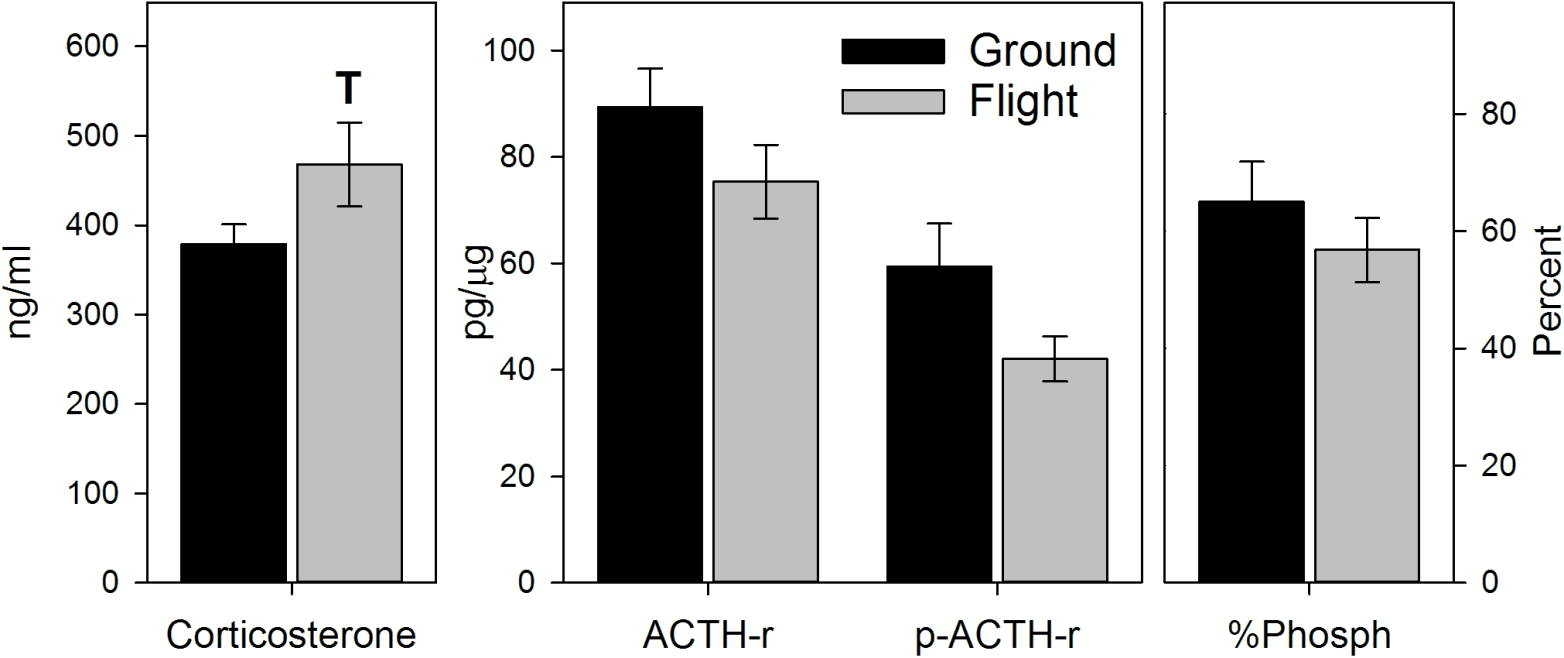
Effects of spaceflight on corticosterone and ACTH receptor levels in the adrenal gland. Corticosterone was quantified via ELISA. Values represent means ± SEM. N = 8 for Ground controls, 5 for Flight. T = trend, P = 0.066. ACTH receptor (ACTH-r) levels quantified via ELISA. p-ACTH-r = phosphorylated ACTH-r. Values represent means ± SEM. N = 11 for Ground controls, 7 for Flight.

We observed a trend for a spaceflight-induced increase in corticosterone levels in the adrenal glands (*P* = 0.066, Fig 2). Although there were consistent decreases noted in ACTH receptors (total or phosphorylated), these also did not reach significance. However, the corticosterone/phosphorylated ACTH receptor ratio was greater in mice who experienced spaceflight (*P<*0.05, See S1 Table).

**Fig 3:**
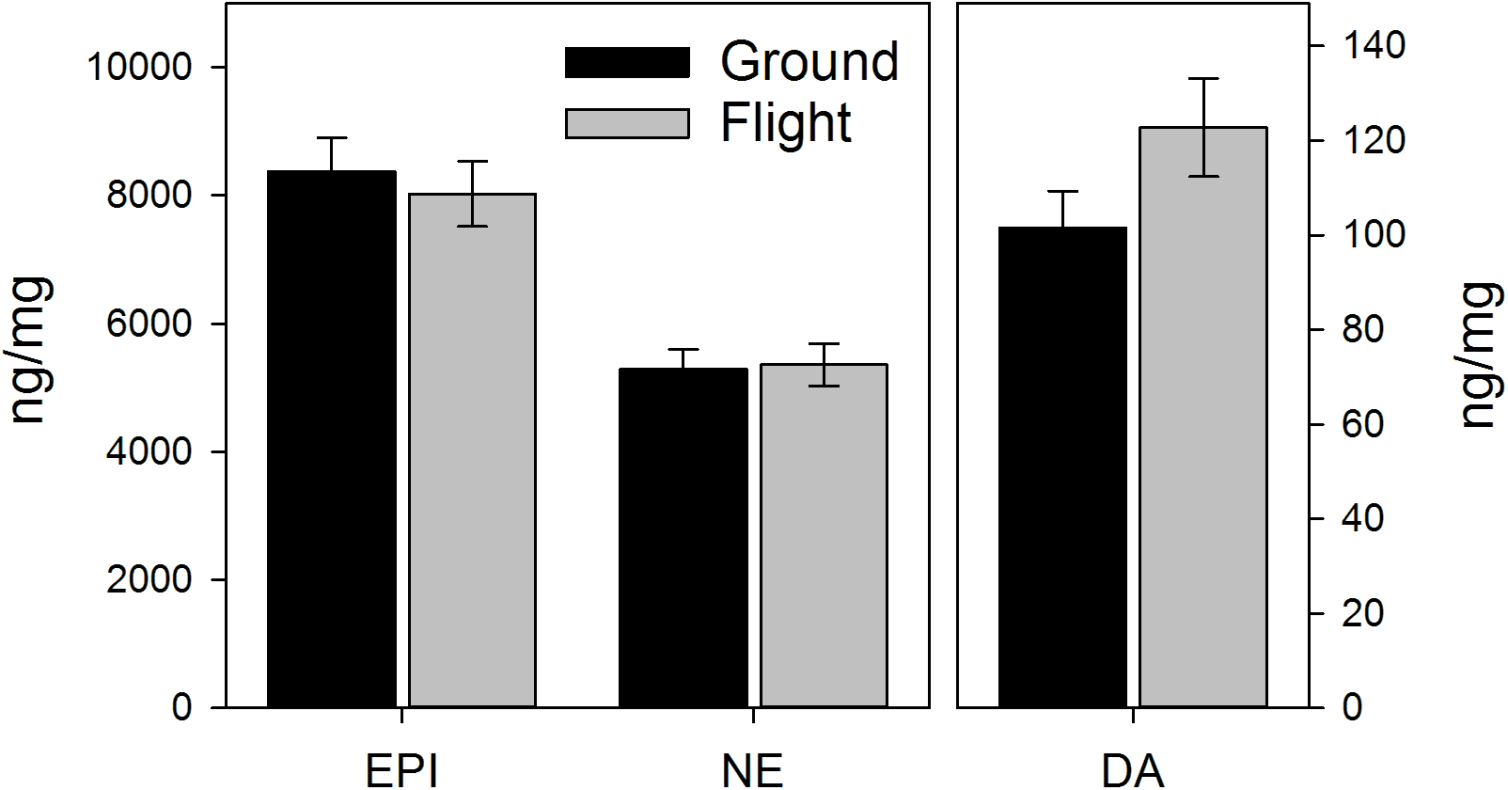
Effects of spaceflight on catecholamine levels in the adrenal gland. Catecholamines measured via HPLC. EPI = epinephrine. NE = Norepinephrine. DA = dopamine. Values represent means ± SEM. N = 13 for Ground controls, 7 for Flight.

There were no significant signs of spaceflight-induced changes in the adrenal catecholamines, norepinephrine and epinephrine (Fig 3). Similarly, although there was a slight elevation in dopamine in flight mice, this was not significant. However, this elevation resulted in significant increases in the dopamine/norepinephrine ratio in spaceflight mice (*P<*0.05, See S1 Table). Spaceflight tended to decrease the norepinephrine/corticosterone and epinephrine/corticosterone ratios (*P* = 0.068 and *P* = 0.056, respectively, S1 Table), but had no significant impact on the dopamine/corticosterone ratio.

### Spaceflight altered liver metabolite levels

We performed untargeted metabolite profiling in the liver, comparing spaceflight and AEM ground controls. The effect of spaceflight on energy and lipid metabolism in these mice has been recently described [35]. Metabolites relevant to the current discussion are included in Table 1. We found significant increases in corticosterone as well as changes in some of the components of glutathione (GSH) synthesis. Specifically, there were decreases in GSH with corresponding increases in 2-aminobutyrate and ophthalmate, potentially indicative of increased oxidative stress. Osmolyte concentrations were altered, with increases in betaine and taurine, and decreases in alanine, glycerophosphorylcholine (GPC) and hypotaurine observed. Finally, we found an increase in glycerol and decreased levels of 3-indoxyl sulfate, a measure of kidney function commonly characterized in the blood.

**Table 1:** Effects of spaceflight on liver metabolomics. Values represent fold changes over AEM controls. All other values are significant (P<0.05). Analysis provided by Metabolon, Inc.

Function	Metabolite	Fold Change
Steroid Synthesis	Corticosterone	1.51
Glutathione (GSH) Synthesis	2-aminobutyrate	1.62
	Cystathionine	-1.39
	Cysteinylglycine (cys-gly)	-1.43
	Glutathione, reduced (GSH)	-1.85
	Ophthalmate	2.33
Osmolyte	Alanine	-1.30
	Betaine	1.83
	Glycerophosphorylcholine (GPC)	-3.57
	Hypotaurine	-1.43
	Taurine	1.17
Kidney	3-indoxyl sulfate	-1.96
Gluconeogenesis	Glycerol	1.42

### Spaceflight altered liver gene expression patterns related to innate immunity, oxidative stress and metabolism

Although we performed a full genomic screen of changes in mouse liver gene expression after spaceflight, we present only a subset of the data here. Similarly, although there are multiple methods for analyzing the data (e.g. basic fold changes, pathway analysis, upstream transcription regulation analysis, etc.), here we focus on genes identified though a functional analysis via IPA. This analysis allowed us to identify genes that are both functionally related (based on the current state of the literature) and statistically correlated to spaceflight. This analysis takes into consideration that, in some cases, down-regulation of a particular gene may actually lead to the activation of a particular function, or *vice versa*.

Table 2 lists a subset of the immune and oxidative stress-related functions identified by IPA to be significantly altered in the liver by spaceflight (z-score >2.0 for activation, <-2.0 for deactivation). Generally, spaceflight appears to have up-regulated functions related to viral infections (z > 4.2) and infections of cells (z > 2.8). Likely linked to this is the activation of clathrin- and receptor-mediated endocytosis (z > 3.1 and z > 2.7, respectively), fusion of late endosomes (z > 2.1) and formation of peroxisomes (z > 2.1).

**Table 2:** Spaceflight effect on immune-related liver gene expression based on functional analysis. Functional analysis was performed with IPA software. Activation z score = statistical value representing the activation state. Z > 2.0 or <-2.0 is generally considered significant. P value = P-value of overlap, a statistical representation describing the level of overlap of genes in our data set compared to the total number of genes known to be directly involved in a particular function based on the literature. # molecules = Number of genes in our data set known to be directly involved in a particular function based on the literature. A complete description of the analysis can be found on the IPA website (http://www.ingenuity.com/).

Function	Activation z-score	Predicted Activation State	p-Value	# Genes
Viral Infection	4.278	Increased	2.34E-06	335
Clathrin-Mediated Endocytosis	3.125	Increased	1.70E-02	20
Infection of Cells	2.838	Increased	5.61E-04	139
Receptor-Mediated Endocytosis	2.775	Increased	2.21E-02	23
Formation of Peroxisomes	2.191	Increased	6.78E-03	6
Fusion of Late Endosomes	2.183	Increased	1.90E-02	6
Activation of CD8+ T Lymphocyte	-2.000	Decreased	7.00E-03	5
Senescence of Cells	-2.555	Decreased	2.96E-02	61
***Trends***				
*Oxidative Stress Response of Cells*	*-1*.*773*	Decreased	*8*.*88E-03*	*18*
*Cell Death of Granulocytes*	*-1*.*794*	Decreased	*3*.*83E-02*	*26*
*Metabolism of Reactive Oxygen Species*	*-1*.*796*	Decreased	*3*.*31E-02*	*106*
*Stress Response of Cells*	*-1*.*948*	Decreased	*1*.*34E-03*	*33*
*Synthesis of Reactive Oxygen Species*	*-1*.*984*	Decreased	*3*.*52E-02*	*104*

Surprisingly, despite the increase in activity related to viral infection, there is a down-regulation in the functional activation of CD8+ T lymphocytes (z < -2.0). Furthermore, although z-scores did not reach the level of significance, there also are trends for the down-regulation of functions related to oxidative stress and ROS metabolism (z < -1.7).

Table 3 describes the functional analyses of genes in the liver related to glycolysis and lipid metabolism. In general, functions related to the generation and sequestration of glucose are up-regulated (z > 2.0) by spaceflight, while functions related to glycolysis are down-regulated (z < -2.0). There are also strong trends for increases in functions that involve the oxidation of lipids (z > 1.8).

**Table 3:** Spaceflight effect on liver expression for genes involved in carbohydrate and lipid metabolism. Functional analysis was performed with IPA software. Activation z score = statistical value representing the activation state. Z > 2.0 or <-2.0 is generally considered significant. P value = P-value of overlap, a statistical representation describing the level of overlap of genes in our data set compared to the total number of genes known to be directly involved in a particular function based on the literature. # molecules = Number of genes in our data set known to be directly involved in a particular function based on the literature. A complete description of the analysis can be found on the IPA website (http://www.ingenuity.com/).

Function	Activation z-score	Predicted Activation State	p-Value	# Genes
Incorporation of D-Glucose	2.000	Increased	3.93E-02	6
Synthesis of Phosphatidylserine	2.000	Increased	1.51E-03	5
Beta-Oxidation of Fatty Acid	2.153	Increased	2.00E-02	17
Concentration of Choline-Phospholipid	2.154	Increased	1.27E-02	14
Synthesis of D-Glucose	2.302	Increased	3.44E-02	21
Synthesis of Monosaccharide	2.463	Increased	2.76E-02	22
Uptake of Fatty Acid	2.754	Increased	1.67E-02	19
Glycolysis of Carbohydrate	-2.758	Decreased	1.36E-02	9
Glycolysis of D-Glucose	-2.571	Decreased	2.91E-02	8
Glycolysis	-2.548	Decreased	8.88E-03	18
Concentration of Fatty Acid	-2.541	Decreased	2.11E-02	69
Disorder of Lipid Metabolism	-2.040	Decreased	9.39E-03	58
***Trends***				
*Oxidation of Lipid*	*1*.*843*	Increased	*1*.*31E-03*	*56*
*Oxidation of Fatty Acid*	*1*.*891*	Increased	*2*.*53E-03*	*44*
*Biliary Excretion of Lipid*	*1*.*963*	Increased	*2*.*16E-02*	*4*
*Hydrolysis of Diacylglycerol*	*1*.*980*	Increased	*2*.*16E-02*	*4*
*Concentration of Phosphatidylcholine*	*1*.*989*	Increased	*2*.*82E-02*	*11*

Microarray fold-changes summarized in Table 4 reveal that expression of regulators of glycogen synthesis, such as glycogen synthase (Gys2), protein phosphatase 1 catalytic subunits (Ppp1ca, Ppp1cb), Gbe1, Foxo1 and Gsk3b were augmented and glycogen phosphorylase (Pygl) mRNA expression was decreased. PEPCK (Pck1) was unchanged. Since insulin is associated with increased glycogen storage, we also performed IPA analysis on expression of gene transcripts in the insulin signaling pathway and found that gene expression levels were decreased in the flight mice for IRS1, IRS2, PI3K and much of the MAPK pathway (S4 Fig).

**Table 4:** mRNA expression changes in the liver for selected genes involved in glycogen metabolism. Fold changes, *P* and q values are calculated using CARMAWeb. *P* < 0.05 is considered significant. The Entrez accession number is given by entrezid.

	entrezid	q	p	FLT/AEM
				(log_2_)
*Foxo1*	56458	3.15E-03	1.26E-04	0.440
*Gbe1*	74185	5.44E-02	2.19E-02	0.405
*Gys2*	232493	9.93E-02	5.94E-02	0.359
*Ppp1cb*	19046	9.51E-02	5.51E-02	0.294
*Ppp1ca*	19045	2.49E-02	6.22E-03	0.259
*Gsk3b*	56637	1.39E-01	1.06E-01	0.148
*Pck1*	18534	3.72E-01	5.93E-01	0.030
*Pygl*	110095	1.01E-02	1.25E-03	-0.204

### Spaceflight mice lose glycogen stores in liver

**Fig 4:**
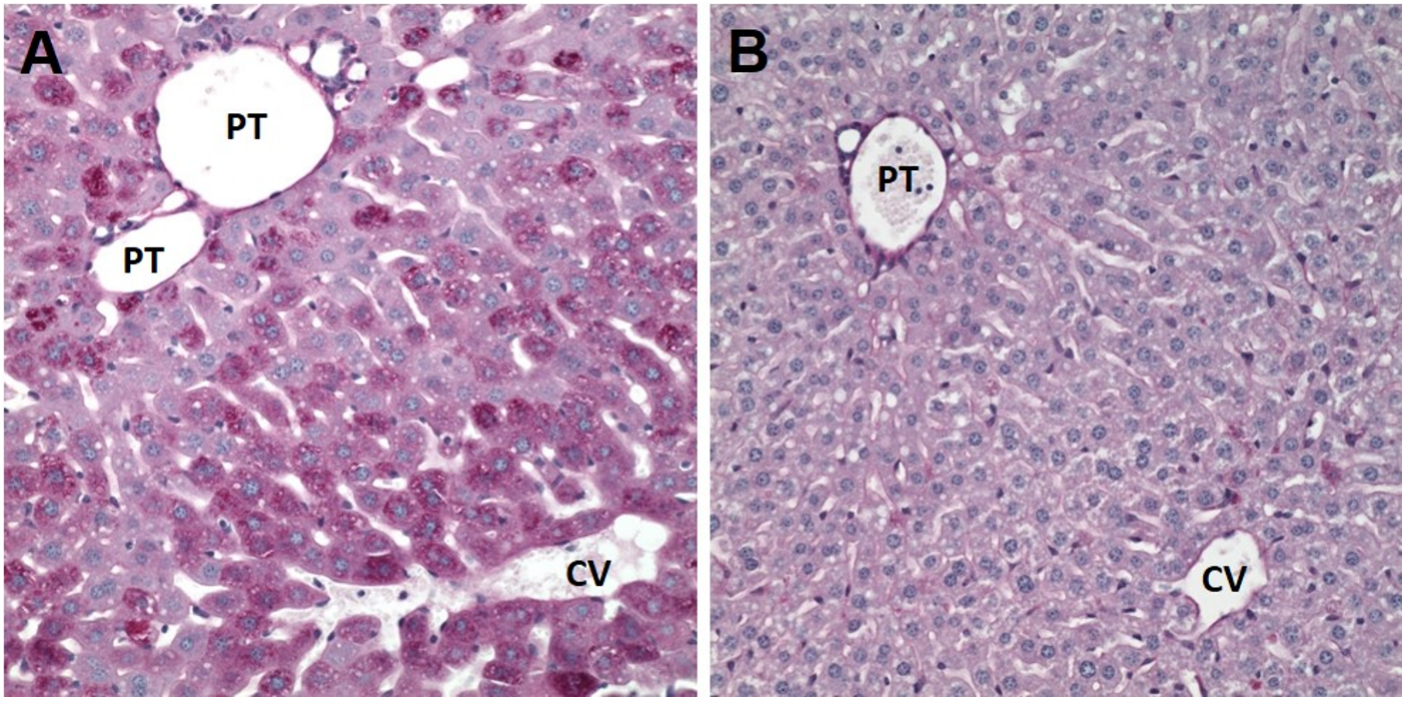
Effects of spaceflight on hepatic glycogen stores. Liver tissue was fixed using 4% paraformaldehyde and stained with Periodic Acid Schiff stain to visualize glycogen. PT = portal triad, CV = central vein. A representative image from *n* = 5 mice per group is shown.

Periodic Acid Schiff (PAS) staining of fixed liver tissue sections was performed on n = 4–5 mice per group. Representative images are shown in Fig 4. Inspection of the stained sections revealed a profound loss of glycogen staining in the flight mice (panel B) as compared with AEM controls (panel A), supporting the microarray results.

## Discussion

Spaceflight appears to have had a significant impact on both immune and metabolomic function. These differences occur in the cell count and proportion data (see S1 Discussion), cell function, metabolites and in gene expression. As we will show, be believe these changes do not occur independently and are, in fact, linked.

There were increases in background ROS levels measured in splenocytes *ex vivo* after flight, suggesting an increase in oxidative stress. High oxidative stress would be consistent with our results from STS-118 where we found increases in the expression of several genes in the liver involved in ROS scavenging and metabolism [5]. This would also be consistent with the increase in the GSSG:GSH ratio (due to the decrease noted in GSH levels) found in metabolomic analysis of the spaceflight livers noted here.

However, although there was an increasing trend noted in another marker of oxidative stress, cysteine-glutathione disulfide, this did not reach the level of significance (Table 1). Furthermore, the increase in GSSG:GSH ratio was due primarily to a decrease in GSH with no corresponding increase in GSSG. While the decrease noted in cysteinyl-glycine (cys-gly) suggests that there may be less GSH breakdown with spaceflight, there were no significant changes in any of the quantified γ-glutamyl amino acids, nor in any other metabolic components of the cycle, including 5-oxoproline, glutamate, cysteine, or glycine. This suggests a more likely explanation for the observed metabolite changes is that there was a decrease in GSH synthesis leading to a lower capacity to deal with oxidative stress.

**Fig 5:**
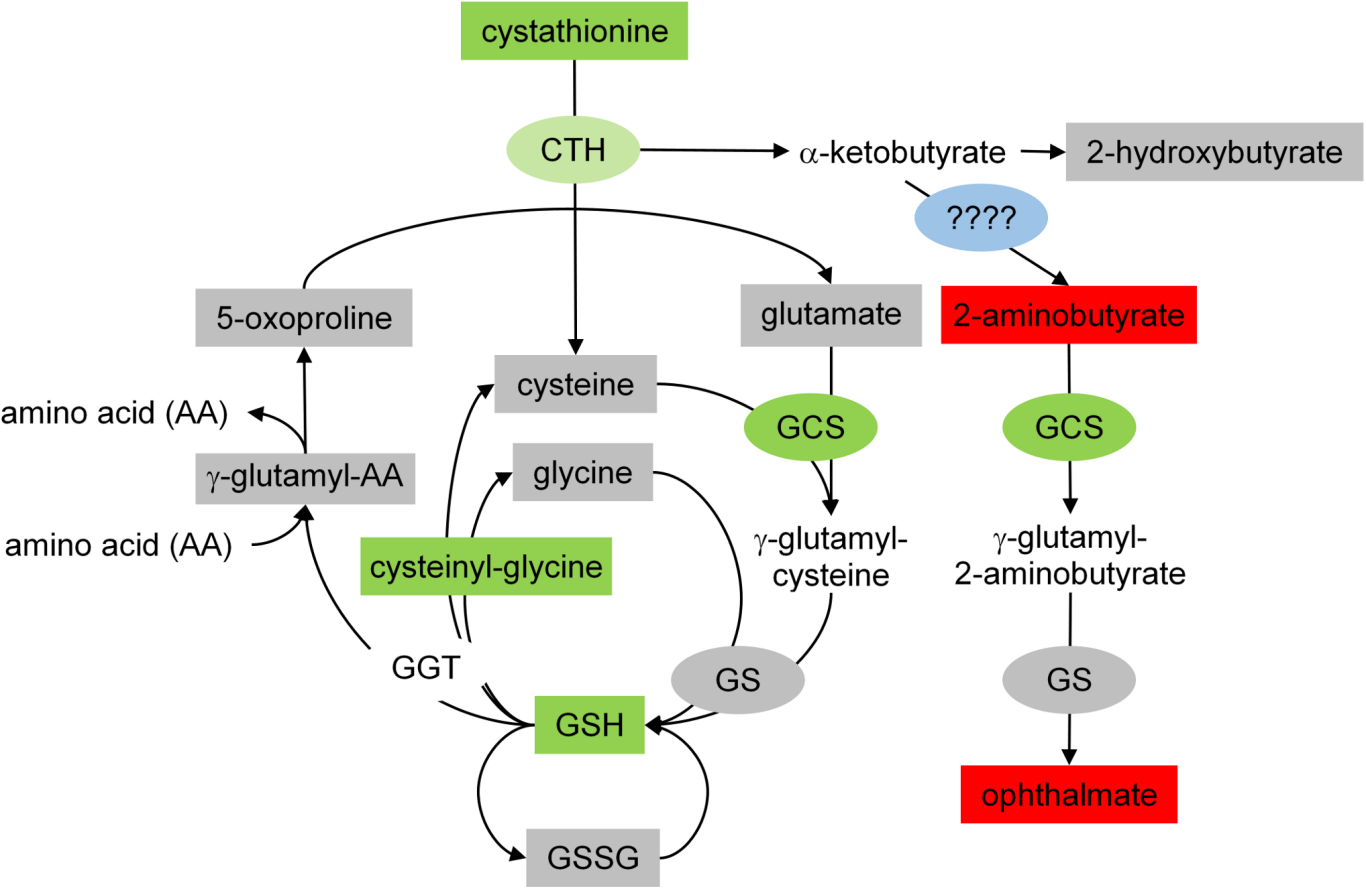
Effects of spaceflight on GSH production. This figure represents a combination of data generated through genomics and metabolomics. CTH = cystathionase. cys-gly = cysteinylglycine. GCS = glutamyl-cysteine synthase. GGT = gamma-glutamyl transpeptidase. GS = glutathione synthase. GSH = glutathione. GSSG = glutathione (oxidized). Rectangles represent metabolomics data. Ovals represent genomics data. Grey = unchanged. Green = down-regulated. Red = up-regulated. Blue = specific transaminase unidentified.

The enzymes responsible for converting glutamate, cysteine, and glycine into GSH are glutamyl-cysteine synthetase (GCS) and glutathione synthetase (GS) (Fig 5). These enzymes are also part of another, separate branch of the pathway responsible for converting cystathionine (a precursor for cysteine) into ophthalmate. This is critical because although cystathionine decreased, both ophthalmate and its upstream precursor, 2-aminobutyrate, significantly increased after flight. This suggests that factors related to spaceflight are driving the kinetics of GCS towards ophthalmate production.

Interestingly, ophthalmate is an endogenous analog of GSH and may be a biomarker for oxidative stress and GSH depletion [48, 49]. Consistent with these reports is the possibility that chronic exposures to the spaceflight environment could lead to a situation where GSH is depleted to such an extent that corresponding increases in downstream products such as GSSG and γ-glutamyl-AA is no longer possible. However, this would not be consistent with our transcriptomics analysis discussed above. Further study is required to clarify these findings.

As indicated in Fig 5, this switch in priorities is further suggested by our liver gene expression analysis. While there was no change in the expression of the GS gene, there was a significant decrease in GCS expression, confirming that GSH synthesis was down-regulated. Although there was a trend for a decrease in the expression of cystathionase, the enzyme for breaking cystathionine into cysteine + α-ketobutyrate, we were not able to identify the specific transaminase responsible for converting α-ketobutyrate into 2-aminobutyrate.

Taken together, these results suggest that although there was an overall decrease in cystathionine, some other mechanism(s) may be driving the remaining resources toward the generation of more ophthalmate. Why this should occur is not entirely clear. However, others have reported that ophthalmate levels are increased in the liver when GSH is depleted and the supply of cysteine to the liver is depleted [48]. The end result is a reduction in the level of GSH available to scavenge ROS.

Despite these changes in individual genes, functional analysis of the liver genes via IPA did not indicate any generalized, consistent increases in overall oxidative stress responses. In fact, hepatocyte-specific oxidative stress response functions all trended toward being slightly down-regulated (Table 2). This suggested that something other than oxidative stress may be going on.

Another possible explanation is that overall transaminase (or aminotransferase) levels increased as a consequence of muscle atrophy. Among other things, transaminases are involved in breaking proteins down into amino acids when blood sugar levels are low, usually at the expense of muscle tissue. This is interesting because spaceflight is known to induce muscle atrophy, in general, and was recently reported to do so in these mice specifically [50]. Unfortunately, we were unable to identify the specific transaminase responsible for converting α-ketobutyrate into 2-aminobutyrate (Fig 5) and cannot verify this hypothesis.

In addition to the increases in background ROS levels in splenocytes, there were large increases in the capacity to generate an oxidative burst in the splenocytes, despite the large decreases in cell counts. Because we did not isolate phagocytic populations for these *ex vivo* assays, the proportion of cells in the spleen after flight is of critical importance in interpreting this result. However, increases in burst activity occurred in spite of *decreases* in granulocyte proportions (and no change in monocyte/macrophages) suggesting that the respiratory burst activity of the cells themselves increased.

There is very little in the literature describing the impact of spaceflight on *ex vivo* burst activity in splenocytes after spaceflight. Kaur *et al*. reported decreases in burst capacity in both monocytes [51] and neutrophils [52] from the blood of astronauts collected immediately after landing. Although we did not have the opportunity to measure oxidative burst capacity in the blood of our mice, this difference in response suggests that either the response is organ-specific and/or primed populations specifically traffic to lymphoid organs, such as the spleen.

An increase in induced respiratory burst activity would be consistent with our liver gene expression data which showed a functional up-regulation in the formation of peroxisomes and the fusion of late endosomes (Table 2). However, as mentioned previously, there was a down-regulation in functions related to ROS metabolism. Interestingly, peroxisomes and late endosomes are also involved in lipid processing and metabolism, suggesting the increases in these cellular components may also reflect a change in gluconeogenesis [53].

Despite the increases in ROS activity, we found decreases in overall phagocytic activity in splenocytes after flight. Because this assay was performed *ex vivo* with a constant number of cells, and because we also saw decreases in some phagocyte proportions (e.g. granulocytes), a possible explanation for this decrease is a change in population distributions. However, the decrease in phagocytic activity (as measured by flow cytometry) was roughly 50% while the decrease in granulocyte percentage was only about 3%. We believe that it is unlikely that such a drastic decrease in phagocytic capacity would be due to such a small decrease in phagocyte proportions. Furthermore, decreases in phagocytic capacity have also been reported in both the macrophages and neutrophils in the blood of astronauts immediately after landing [51, 52].

One possible explanation for the changes in immunocyte distribution and function is psychological/physiological stress. In support of this is the trend for a spaceflight-induced up-regulation of adrenal levels of corticosterone, an indicator of increased hypothalamus-pituitary-adrenal (HPA) activity. Metabolomic analysis of both the liver (presented here) and skin [54] of these mice showed similar increases in corticosterone levels. This is also generally consistent with reports of post-flight increases in circulating corticosterone levels in rodent models [7, 17, 26, 55]. A very recent study showed that cortisol levels generally increased in astronaut saliva in-flight compared to pre-flight levels, but this was only a trend [56]. Surprisingly, cortisol levels typically do not change in the blood of astronauts post-flight [31, 32, 57, 58]. There are even at least two reports of *decreases* in plasma cortisol levels in astronauts due to flight [59, 60]. In contrast, urinary cortisol levels typically increased after flight [31, 32, 57–61], but not always [51].

Since corticosterone is synthesized and released in the adrenal cortex and most endocrine tissues have tightly regulated homeostatic mechanisms that keep tissue levels within small discrete ranges (i.e., the amount of hormone being made is the same amount as that being released–allostasis), measuring adrenal corticosterone is not equivalent to measuring it in the blood or other organs. This suggests that the small difference in adrenal corticosterone levels between treatment groups reported here, despite fairly consistent increases found in other tissues, may actually be due to an increase in corticosterone synthesis in the adrenal cortex to compensate for increased corticosterone secretion into the circulation.

Decreases in spleen and thymus mass have long been associated with chronically elevated levels of corticosterone and stress [62–64]. We previously reported consistent decreases in spleen and thymus mass in these mice [1], as well as in rodents flown on STS-77 [18], -108 [2] and -118 [5]. While others have also reported decreases in spleen mass after flight [7, 9, 12, 14, 17], there is at least one report of an increase [55]. Similarly, thymus mass has generally been shown to decrease after flight [9, 12, 17], but there are some reports of increases [17, 55].

If spaceflight-subjected mice were indeed stressed as other findings here and in the literature suggest, particularly during the landing, then this should be reflected in ACTH activity. Adrenal corticosterone secretion is known to be up-regulated by ACTH receptor ligation on adrenocortical cells (predominantly in the *zona fasciculata*). An increase in corticosterone should be coincident with an increase in ACTH levels. Indeed, while plasma ACTH levels did not change in the blood of astronauts after flight [32, 58, 60], urinary ACTH levels increased [32].

Although we did not characterize adrenal ACTH levels directly, we did measure ACTH receptors. Because ACTH receptors are unique G protein-coupled receptors in that ligand activation up-regulates their expression [65–68], this should provide an indirect measure of ACTH activity and HPA activation. However, we did not find significant effects of spaceflight on ACTH receptor expression. Similarly, the percentage of phosphorylated ACTH receptors in the adrenals did not differ between treatment groups after flight. This would seem to suggest that the chronic exposure to the conditions of spaceflight promotes receptor desensitization and suggests that spaceflight may actually reduce the sensitivity to stress.

Adrenal catecholamines are mediators of stress-induced activation of the sympathoadrenomedullary (SAM) axis, and like corticosterone, their production and secretion into the circulation typically increases in response to stress. However, despite the noted increases in corticosterone, we found no changes in adrenal norepinephrine and epinephrine levels. There is very little published in the literature involving activation of the SAM axis after spaceflight in rodent models. However, norepinephrine levels in the kidney of rats either do not change [69] or actually decreased [70] after spaceflight. Interestingly, urinary epinephrine levels did not change [31, 51, 52] or increased [32, 57, 60, 61] in astronauts after flight. Similarly, urinary norepinephrine levels either did not change [51, 52] or increased [31, 32, 57, 60]. Because urinary catecholamine levels are usually assessed using pooled samples collected over a 24-hour period post-landing, we believe that the reported increases are reflective of a response to the landing and not the actual spaceflight environment.

These data suggest that while there may have been an activation of stress pathways after spaceflight, the response was relatively mild and likely did not involve both HPA and SNS activation. Although speculative, given the lack of a significant catecholamine response, it is possible that this elevation in corticosterone is not directly related to psychological stress at all. But rather, the change is related to energy balance and lipid metabolism. As previously stated, spaceflight has long been suspected to alter energy metabolism [71] and stress hormones like glucocorticoids (i.e. corticosterone) are known to play a significant role in metabolism [72, 73]. Indeed, corticosterone promotes fat breakdown in adipose and muscle tissue to provide glycerol to the liver for gluconeogenesis. Consistent with this idea is the fact that the metabolite, glycerol, was also more abundant in flight liver relative to ground controls.

Interestingly, peroxisome proliferator-activated receptor gamma (PPAR) regulated activity increased significantly in the liver after flight. This is important because although the primary function of PPARs is to regulate lipid metabolism, PPARs can also negatively interfere with immune regulators including NF-κB, STAT, and AP-1 signaling pathways [74–79]. PPARs also mediate macrophage responses in the arterial cell wall [76, 77, 80, 81], both by interfering with chemoattraction and adhesion of immunocytes, including T cells and monocytes, and by down-regulating MCP-1 [82], RANTES [83], and vascular cell adhesion molecule-1 (VCAM-1) [75, 84]. We have demonstrated that lipid metabolism and PPAR activity are altered in spaceflight [35]. Given that many of the functional changes we see in immune response overlap considerably with energy/lipid metabolism, we believe that the two phenomena may actually be related.

We observed a profound loss of glycogen stores in the livers of Flight mice. Paradoxically, most regulators of glycogen synthesis were increased, concomitant with a decrease in glycogen phosphorylase mRNA expression, pointing to upregulation of glycogenic rather than glycogenolytic pathways. This may be due, in part, to activation of compensatory mechanisms for restoring normal levels of glycogen stores. PEPCK (Pck1) mRNA expression was unchanged; therefore gluconeogenesis as a mechanism for restoring glycogen was likely not activated. We did not observe changes in hepatic glucose or glucose-6-phosphate concentrations and unfortunately did not have access to blood to measure serum glucose or insulin levels. However, the intracellular downregulation of IRS, PI3K and MAPK pathway genes are classical post-receptor manifestations of insulin resistance that may be induced by lipotoxicity, as we have previously shown [35]. Taken together, our data support the concept of spaceflight-induced alterations in the response to insulin that may correlate with insulin resistance. It will be important to obtain serum in future studies to measure insulin levels in mice and further pursue this link.

In conclusion, spaceflight clearly causes transient changes in immune function. However, the cause may not be exclusively due to psychological or oxidative stress. But rather, the shifts in immune function may be at least partially due to shifts in energy needs in space. Onset of leukocyte regeneration after landing could, of course, also have a significant impact on functional status. As a full description of the impact of spaceflight on lipid metabolism is beyond the scope of this paper, the details of this have been covered elsewhere [35]. However, gene expression functional analysis indicated increases in functions related to glucose synthesis and sequestration, with corresponding decreases in glycolysis functions. Furthermore, the *ex vivo* increases in ROS activity, combined with corresponding increases in peroxisome and endosome-related gene expression, are likely related to spaceflight-induced shifts in lipid metabolism. This further suggests that spaceflight causes a shift in priorities in phagocytic populations away from antigen processing toward lipid processing, potentially increasing risk for infection.

## Supporting information

S1 Fig Proposed model linking spaceflight-induced disruptions in innate immune function and metabolism. (TIF)

S2 Fig Effects of spaceflight on splenic leukocyte populations. Data were obtained using an automated hematology analyzer. Values were normalized to daily Vivarium controls. WBC = white blood cells. LYM = lymphocytes. MON = monocyte/macrophages. GRA = granulocytes. Values represent means ± SEM. N = 8 for Ground controls housed in animal enclosure modules, 5 for Flight. *P<0.001.
(TIF)

S3 Fig Effects of spaceflight on lymphocyte subsets. Values represent means ± SEM. N = 8 for Ground controls, 5 for Flight. Values were normalized to daily Vivarium controls. *P<0.05, **P<0.005, ***P<0.001.
(TIF)

S4 Fig Impact of spaceflight on the insulin signaling pathway. Analysis performed using Ingenuity Pathway Analysis (Qiagen, Inc., Redwood City, CA). Grey = unchanged. Green = down-regulated. Red = up-regulated.
(TIF)

S1 Table Effect of spaceflight on hormone ratios. Values represent means ± SEM. N = 13 for Ground controls, 7 for Flight. NS = Not significant.
(DOCX)

S1 Discussion Effect of spaceflight on splenic immunocyte population distributions. We have analyzed splenic immunocyte populations on three separate space shuttle missions with very similar flight profiles. This additional discussion includes historical context as well as comments on repeatability.
(DOCX)

## References

[1] Gridley DS, Mao XW, Stodieck LS, Ferguson VL, Bateman TA, Moldovan M, et al Changes in mouse thymus and spleen after return from the STS-135 mission in space. PloS one. 2013;8(9):e75097 PubMed Central PMCID: PMC3777930. doi: 10.1371/journal.pone.0075097 24069384 10.1371/journal.pone.0075097 PMC3777930

[2] Gridley DS, Nelson GA, Peters LL, Kostenuik PJ, Bateman TA, Morony S, et al Genetic Models in Applied Physiology: Selected Contribution: Effects of spaceflight on immunity in the C57BL/6 mouse. II. Activation, cytokines, erythrocytes, and platelets. Journal of applied physiology. 2003;94(5):2095–103. doi: 10.1152/japplphysiol.01053.2002 12506046 10.1152/japplphysiol.01053.2002

[3] Gridley DS, Slater JM, Luo-Owen X, Rizvi A, Chapes SK, Stodieck LS, et al Spaceflight effects on T lymphocyte distribution, function and gene expression. Journal of applied physiology. 2009;106(1):194–202. Epub 2008/11/08. doi: 10.1152/japplphysiol.91126.2008 18988762 10.1152/japplphysiol.91126.2008 PMC2636934

[4] Pecaut MJ, Nelson GA, Peters LL, Kostenuik PJ, Bateman TA, Morony S, et al Genetic Models in Applied Physiology: Selected Contribution: Effects of spaceflight on immunity in the C57BL/6 mouse. I. Immune population distributions. Journal of applied physiology. 2003;94(5):2085–94. doi: 10.1152/japplphysiol.01052.2002 12514166 10.1152/japplphysiol.01052.2002

[5] Baqai FP, Gridley DS, Slater JM, Luo-Owen X, Stodieck LS, Ferguson V, et al Effects of spaceflight on innate immune function and antioxidant gene expression. J Appl Physiol (1985). 2009;106(6):1935–42. PubMed Central PMCID: PMC2692779. 19342437 10.1152/japplphysiol.91361.2008 PMC2692779

[6] Ortega MT, Pecaut MJ, Gridley DS, Stodieck LS, Ferguson V, Chapes SK. Shifts in bone marrow cell phenotypes caused by spaceflight. Journal of applied physiology. 2009;106(2):548–55. Epub 2008/12/06. PubMed Central PMCID: PMC2644250. doi: 10.1152/japplphysiol.91138.2008 19056998 10.1152/japplphysiol.91138.2008 PMC2644250

[7] Durnova GN, Kaplansky AS, Portugalov VV. Effect of a 22-day space flight on the lymphoid organs of rats. Aviation, space, and environmental medicine. 1976;47(6):588–91. 938393

[8] Grindeland RE, Popova IA, Vasques M, Arnaud SB. COSMOS 1887 mission overview: effects of microgravity on rat body and adrenal weights and plasma constituents. FASEB journal: official publication of the Federation of American Societies for Experimental Biology. 1990;4:105–9. 2295371 10.1096/fasebj.4.1.2295371

[9] Jahns G, Meylor J, Fast T, Hawes N, Zarow G, editors. Rodent Growth, Behavior, and Physiology Resulting from Flight on the Space Life Sciences-1 Mission. 43rd Congressthe Int Astronautical Federation; 1992 August 28-September 5, 1992; Washington, DC: International Astronautical Federation.

[10] Allebban Z, Gibson LA, Lange RD, Jago TL, Strickland KM, Johnson DL, et al Effects of spaceflight on rat erythroid parameters. Journal of applied physiology. 1996;81(1):117–22. 8828653 10.1152/jappl.1996.81.1.117

[11] Udden MM, Driscoll TB, Gibson LA, Patton CS, Pickett MH, Jones JB, et al Blood volume and erythropoiesis in the rat during spaceflight. Aviation, space, and environmental medicine. 1995;66(6):557–61. 7646406

[12] Congdon CC, Allebban Z, Gibson LA, Kaplansky A, Strickland KM, Jago TL, et al Lymphatic tissue changes in rats flown on Spacelab Life Sciences-2. Journal of applied physiology. 1996;81(1):172–7. 8828660 10.1152/jappl.1996.81.1.172

[13] Serova LV. Weightlessness effects on resistance and reactivity of animals. Physiologist. 1980;23:S22–S6. 7243930

[14] Grove DS, Pishak SA, Mastro AM. The effect of a 10-day space flight on the function, phenotype, and adhesion molecule expression of splenocytes and lymph node lymphocytes. Experimental cell research. 1995;219(1):102–9. doi: 10.1006/excr.1995.1210 7543050 10.1006/excr.1995.1210

[15] Nash PV, Mastro AM. Variable lymphocyte responses in rats after space flight. Experimental cell research. 1992;202(1):125–31. 1511727 10.1016/0014-4827(92)90411-z

[16] Wronski TJ, Li M, Shen Y, Miller SC, Bowman BM, Kostenuik P, et al Lack of effect of spaceflight on bone mass and bone formation in group-housed rats. Journal of applied physiology. 1998;85(1):279–85. 9655787 10.1152/jappl.1998.85.1.279

[17] Chapes SK, Simske SJ, Sonnenfeld G, Miller ES, Zimmerman RJ. Effects of space flight and PEG-IL-2 on rat physiological and immunological responses. Journal of applied physiology. 1999;86(6):2065–76. 10368375 10.1152/jappl.1999.86.6.2065

[18] Pecaut MJ, Simske SJ, Fleshner M. Spaceflight induces changes in splenocyte subpopulations: effectiveness of ground-based models. Am J Physiol Regulatory Integrative Comp Physiol. 2000;279(6):R2072–R8. 10.1152/ajpregu.2000.279.6.R2072 11080071

[19] Allebban Z, Ichiki AT, Gibson LA, Jones JB, Congdon CC, Lange RD. Effects of spaceflight on the number of rat peripheral blood leukocytes and lymphocyte subsets. J Leukoc Biol. 1994;55(2):209–13. 8301218 10.1002/jlb.55.2.209

[20] Sonnenfeld G, Mandel AD, Konstantinova IV, Berry WD, Taylor GR, Lesnyak AT, et al Spaceflight alters immune cell function and distribution. Journal of applied physiology. 1992;73(2 Suppl):191S–5S. 1526951 10.1152/jappl.1992.73.2.S191

[21] Ichiki AT, Gibson LA, Jago TL, Strickland KM, Johnson DL, Lange RD, et al Effects of spaceflight on rat peripheral blood leukocytes and bone marrow progenitor cells. J Leukoc Biol. 1996;60(1):37–43. 8699121 10.1002/jlb.60.1.37

[22] Sonnenfeld G, Foster M., Morton D, Bailliard F, Fowler NA, Hakenewerth AM, et al Spaceflight and development of immune responses. Journal of applied physiology. 1998;85(4):1429–33. 9760337 10.1152/jappl.1998.85.4.1429

[23] Sonnenfeld G. Immune responses in space flight. International journal of sports medicine. 1998;19(Suppl 3):S195–S202. 9722285 10.1055/s-2007-971992

[24] Meehan RT, Neale LS, Kraus ET, Stuart CA, Smith ML, Cintron NM, et al Alteration in human mononuclear leucocytes following space flight. Immunol. 1992;76:491–7. PMC1421684 1326479

[25] Berry CA. Summary of medical experience in the Apollo 7 through 11 manned spaceflights. Aerosp Med. 1970;41(5):500–19. 4393427

[26] Lesnyak AT, Sonnenfeld G, Rykova MP, Meshkov DO, Mastro A, Konstantinova I. Immune changes in test animals during spaceflight. J Leukoc Biol. 1993;54(3):214–26. 8371051 10.1002/jlb.54.3.214

[27] Merrill AH, Wang E, Mullins RE, Grindeland RE, Popova IA. Analyses of plasma for metabolic and hormonal changes in rats flown aboard COSMOS 2044. Journal of applied physiology. 1992;73(2 Suppl):132S–5S. 1526939 10.1152/jappl.1992.73.2.S132

[28] Meehan R, Whitson P, Sams C. The role of psychoneuroendocrine factors on spaceflight-induced immunological alterations. J Leukoc Biol. 1993;54(3):236–44. 8371053 10.1002/jlb.54.3.236

[29] Blanc S, Somody L, Gharib A, Gauquelin G, Gharib C, Sarda N. Counteraction of spaceflight-induced changes in the rat central serotonergic system by adrenalectomy and corticosteroid replacement. Neurochem Int. 1998;33(4):375–82. 9840229 10.1016/s0197-0186(98)00042-4

[30] Stowe RP, Mehta SK, Ferrando AA, Feeback DL, Pierson DL. Immune responses and latent herpesvirus reactivation in spaceflight. Aviation, space, and environmental medicine. 2001;72(10):884–91. 11601551

[31] Stowe RP, Pierson DL, Barrett AD. Elevated stress hormone levels relate to Epstein-Barr virus reactivation in astronauts. Psychosomatic medicine. 2001;63(6):891–5. 11719627 10.1097/00006842-200111000-00007

[32] Stowe RP, Sams CF, Mehta SK, Kaur I, Jones ML, Feeback DL, et al Leukocyte subsets and neutrophil function after short-term spaceflight. J Leukoc Biol. 1999;65(2):179–86. 10088600 10.1002/jlb.65.2.179

[33] Stein TP, Schluter MD. Excretion of IL-6 by astronauts during spaceflight. The American journal of physiology. 1994;266(3 Pt 1):E448–52. 8166266 10.1152/ajpendo.1994.266.3.E448

[34] Da Silva MS, Zimmerman PM, Meguid MM, Nandi J, Ohinata K, Xu Y, et al Anorexia in space and possible etiologies: an overview. Nutrition. 2002;18(10):805–13. 12361771 10.1016/s0899-9007(02)00915-2

[35] Jonscher KR, Alfonso-Garcia A, Suhalim JL, Orlicky DJ, Potma EO, Ferguson VL, et al Spaceflight activates lipotoxic pathways in mouse liver. PloS one. 2016;11(4):e0152877 doi: 10.1371/journal.pone.0152877 27097220 10.1371/journal.pone.0152877 PMC4838331

[36] Peckett AJ, Wright DC, Riddell MC. The effects of glucocorticoids on adipose tissue lipid metabolism. Metabolism: clinical and experimental. 2011;60(11):1500–10. 21864867 10.1016/j.metabol.2011.06.012

[37] Biswas SK, Mantovani A. Orchestration of metabolism by macrophages. Cell metabolism. 2012;15(4):432–7. doi: 10.1016/j.cmet.2011.11.013 22482726 10.1016/j.cmet.2011.11.013

[38] Lumeng CN, Bodzin JL, Saltiel AR. Obesity induces a phenotypic switch in adipose tissue macrophage polarization. The Journal of clinical investigation. 2007;117(1):175–84. doi: 10.1172/JCI29881 17200717 10.1172/JCI29881 PMC1716210

[39] Evans AM, DeHaven CD, Barrett T, Mitchell M, Milgram E. Integrated, nontargeted ultrahigh performance liquid chromatography/electrospray ionization tandem mass spectrometry platform for the identification and relative quantification of the small-molecule complement of biological systems. Analytical chemistry. 2009;81(16):6656–67. doi: 10.1021/ac901536h 19624122 10.1021/ac901536h

[40] Weiner J 3rd, Parida SK, Maertzdorf J, Black GF, Repsilber D, Telaar A, et al Biomarkers of inflammation, immunosuppression and stress with active disease are revealed by metabolomic profiling of tuberculosis patients. PloS one. 2012;7(7):e40221 PubMed Central PMCID: PMC3402490. doi: 10.1371/journal.pone.0040221 22844400 10.1371/journal.pone.0040221 PMC3402490

[41] Lawton KA, Berger A, Mitchell M, Milgram KE, Evans AM, Guo L, et al Analysis of the adult human plasma metabolome. Pharmacogenomics. 2008;9(4):383–97. doi: 10.2217/14622416.9.4.383 18384253 10.2217/14622416.9.4.383

[42] Sha W, da Costa KA, Fischer LM, Milburn MV, Lawton KA, Berger A, et al Metabolomic profiling can predict which humans will develop liver dysfunction when deprived of dietary choline. FASEB journal: official publication of the Federation of American Societies for Experimental Biology. 2010;24(8):2962–75. PubMed Central PMCID: PMC2909293. 20371621 10.1096/fj.09-154054 PMC2909293

[43] Dehaven CD, Evans AM, Dai H, Lawton KA. Organization of GC/MS and LC/MS metabolomics data into chemical libraries. Journal of cheminformatics. 2010;2(1):9 PubMed Central PMCID: PMC2984397. doi: 10.1186/1758-2946-2-9 20955607 10.1186/1758-2946-2-9 PMC2984397

[44] Storey JD, Tibshirani R. Statistical significance for genomewide studies. Proceedings of the National Academy of Sciences of the United States of America. 2003;100(16):9440–5. PubMed Central PMCID: PMCPMC170937. doi: 10.1073/pnas.1530509100 12883005 10.1073/pnas.1530509100 PMC170937

[45] Rainer J, Sanchez-Cabo F, Stocker G, Sturn A, Trajanoski Z. CARMAweb: comprehensive R- and bioconductor-based web service for microarray data analysis. Nucleic Acids Res. 2006;34(Web Server issue):W498–503. PubMed Central PMCID: PMCPMC1538903. doi: 10.1093/nar/gkl038 16845058 10.1093/nar/gkl038 PMC1538903

[46] Russell TD, Palmer CA, Orlicky DJ, Fischer A, Rudolph MC, Neville MC, et al Cytoplasmic lipid droplet accumulation in developing mammary epithelial cells: roles of adipophilin and lipid metabolism. J Lipid Res. 2007;48(7):1463–75. doi: 10.1194/jlr.M600474-JLR200 17452747 10.1194/jlr.M600474-JLR200

[47] Rich L, Whittaker P. Collagen and picrosirius red staining: A polarized light assessment of fibrilar hue and spatial distribution. Braz J Morphol Sci. 2005;22:97–104.

[48] Dello SA, Neis EP, de Jong MC, van Eijk HM, Kicken CH, Olde Damink SW, et al Systematic review of ophthalmate as a novel biomarker of hepatic glutathione depletion. Clinical nutrition. 2013;32(3):325–30. Epub 2012/11/28. doi: 10.1016/j.clnu.2012.10.008 23182341 10.1016/j.clnu.2012.10.008

[49] Soga T, Baran R, Suematsu M, Ueno Y, Ikeda S, Sakurakawa T, et al Differential metabolomics reveals ophthalmic acid as an oxidative stress biomarker indicating hepatic glutathione consumption. The Journal of biological chemistry. 2006;281(24):16768–76. doi: 10.1074/jbc.M601876200 16608839 10.1074/jbc.M601876200

[50] Sung M, Li J, Spieker AJ, Spatz J, Ellman R, Ferguson VL, et al Spaceflight and hind limb unloading induce similar changes in electrical impedance characteristics of mouse gastrocnemius muscle. Journal of musculoskeletal & neuronal interactions. 2013;13(4):405–11. 24292610 PMC4653813

[51] Kaur I, Simons ER, Castro VA, Ott CM, Pierson DL. Changes in monocyte functions of astronauts. Brain, behavior, and immunity. 2005;19(6):547–54. doi: 10.1016/j.bbi.2004.12.006 15908177 10.1016/j.bbi.2004.12.006

[52] Kaur I, Simons ER, Castro VA, Mark Ott C, Pierson DL. Changes in neutrophil functions in astronauts. Brain, behavior, and immunity. 2004;18(5):443–50. doi: 10.1016/j.bbi.2003.10.005 15265537 10.1016/j.bbi.2003.10.005

[53] Kersten S. Integrated physiology and systems biology of PPARalpha. Molecular metabolism. 2014;3(4):354–71. PubMed Central PMCID: PMC4060217. doi: 10.1016/j.molmet.2014.02.002 24944896 10.1016/j.molmet.2014.02.002 PMC4060217

[54] Mao XW, Pecaut MJ, Stodieck LS, Ferguson VL, Bateman TA, Bouxsein ML, et al Biological and metabolic response in STS-135 space-flown mouse skin. Free radical research. 2014;48(8):890–7. doi: 10.3109/10715762.2014.920086 24796731 10.3109/10715762.2014.920086

[55] Chapes SK, Simske SJ, Forsman AD, Bateman TA, Zimmerman RJ. Effects of space flight and IGF-1 on immune function. Advances in space research: the official journal of the Committee on Space Research. 1999;23(12):1955–64. 10.1016/s0273-1177(99)00456-1 11710377

[56] Mehta SK, Laudenslager ML, Stowe RP, Crucian BE, Sams CF, Pierson DL. Multiple latent viruses reactivate in astronauts during Space Shuttle missions. Brain, behavior, and immunity. 2014. 10.1016/j.bbi.2014.05.014 24886968

[57] Pierson DL, Stowe RP, Phillips TM, Lugg DJ, Mehta SK. Epstein-Barr virus shedding by astronauts during space flight. Brain, behavior, and immunity. 2005;19(3):235–42. doi: 10.1016/j.bbi.2004.08.001 15797312 10.1016/j.bbi.2004.08.001

[58] Mehta SK, Kaur I, Grimm EA, Smid C, Feeback DL, Pierson DL. Decreased non-MHC-restricted (CD56+) killer cell cytotoxicity after spaceflight. J Appl Physiol (1985). 2001;91(4):1814–8. 11568167 10.1152/jappl.2001.91.4.1814

[59] Leach CS, Rambaut PC. Endocrine responses in long-duration manned space flight. Acta astronautica. 1975;2(1–2):115–27. 11841088 10.1016/0094-5765(75)90048-x

[60] Mehta SK, Stowe RP, Feiveson AH, Tyring SK, Pierson DL. Reactivation and shedding of cytomegalovirus in astronauts during spaceflight. The Journal of infectious diseases. 2000;182(6):1761–4. doi: 10.1086/317624 11069250 10.1086/317624

[61] Stowe RP, Pierson DL, Feeback DL, Barrett AD. Stress-induced reactivation of Epstein-Barr virus in astronauts. Neuroimmunomodulation. 2000;8(2):51–8. doi: 26453 10965229 10.1159/000026453

[62] Voorhees JL, Powell ND, Moldovan L, Mo X, Eubank TD, Marsh CB. Chronic restraint stress upregulates erythropoiesis through glucocorticoid stimulation. PloS one. 2013;8(10):e77935 PubMed Central PMCID: PMC3799740. doi: 10.1371/journal.pone.0077935 24205034 10.1371/journal.pone.0077935 PMC3799740

[63] Batuman OA, Sajewski D, Ottenweller JE, Pitman DL, Natelson BH. Effects of repeated stress on T cell numbers and function in rats. Brain, behavior, and immunity. 1990;4(2):105–17. 2393722 10.1016/0889-1591(90)90013-g

[64] Zoladz PR, Conrad CD, Fleshner M, Diamond DM. Acute episodes of predator exposure in conjunction with chronic social instability as an animal model of post-traumatic stress disorder. Stress. 2008;11(4):259–81. PubMed Central PMCID: PMC2535807. doi: 10.1080/10253890701768613 18574787 10.1080/10253890701768613 PMC2535807

[65] Morita TM, Imai T, Murata Y, Kambe F, Funahashi H, Takagi H, et al Adrenocorticotropic hormone (ACTH) increases the expression of its own receptor gene. Endocrine journal. 1995;42(4):475–80. 8556053 10.1507/endocrj.42.475

[66] Penhoat A, Jaillard C, Saez JM. Regulation of bovine adrenal cell corticotropin receptor mRNA levels by corticotropin (ACTH) and angiotensin-II (A-II). Molecular and cellular endocrinology. 1994;103(1–2):R7–10. 7958385 10.1016/0303-7207(94)90088-4

[67] Penhoat A, Jaillard C, Saez JM. Corticotropin positively regulates its own receptors and cAMP response in cultured bovine adrenal cells. Proceedings of the National Academy of Sciences of the United States of America. 1989;86(13):4978–81. PubMed Central PMCID: PMC297539. 2544885 10.1073/pnas.86.13.4978 PMC297539

[68] Lebrethon MC, Jaillard C, Naville D, Begeot M, Saez JM. Regulation of corticotropin and steroidogenic enzyme mRNAs in human fetal adrenal cells by corticotropin, angiotensin-II and transforming growth factor beta 1. Molecular and cellular endocrinology. 1994;106(1–2):137–43. 7895901 10.1016/0303-7207(94)90195-3

[69] Fagette S, Somody L, Koubi H, Fareh J, Viso M, Gharib C, et al Central and peripheral noradrenergic responses to 14 days of spaceflight (SLS-2) or hindlimb suspension in rats. Aviation, space, and environmental medicine. 1996;67(5):458–62. 8725473

[70] Fareh J, Cottet-Emard JM, Pequignot JM, Jahns G, Meylor J, Viso M, et al Norepinephrine content in discrete brain areas and neurohypophysial vasopressin in rats after a 9-d spaceflight (SLS-1). Aviation, space, and environmental medicine. 1993;64(6):507–11. 8338496

[71] Buckey J, J. C. Chapter 8: Nutritian: Maintaining Body Mass and Preventing Disease. Space Physiology. New York: Oxford University Press; 2006.

[72] Dallman MF. Modulation of stress responses: how we cope with excess glucocorticoids. Experimental neurology. 2007;206(2):179–82. PubMed Central PMCID: PMC2795792. doi: 10.1016/j.expneurol.2007.06.002 17628543 10.1016/j.expneurol.2007.06.002 PMC2795792

[73] Dallman MF, Akana SF, Pecoraro NC, Warne JP, la Fleur SE, Foster MT. Glucocorticoids, the etiology of obesity and the metabolic syndrome. Current Alzheimer research. 2007;4(2):199–204. 17430247 10.2174/156720507780362236

[74] Delerive P, Martin-Nizard F, Chinetti G, Trottein F, Fruchart JC, Najib J, et al Peroxisome proliferator-activated receptor activators inhibit thrombin-induced endothelin-1 production in human vascular endothelial cells by inhibiting the activator protein-1 signaling pathway. Circulation research. 1999;85(5):394–402. 10473669 10.1161/01.res.85.5.394

[75] Marx N, Sukhova GK, Collins T, Libby P, Plutzky J. PPARalpha activators inhibit cytokine-induced vascular cell adhesion molecule-1 expression in human endothelial cells. Circulation. 1999;99(24):3125–31. 10377075 10.1161/01.cir.99.24.3125 PMC4231776

[76] Chinetti G, Griglio S, Antonucci M, Torra IP, Delerive P, Majd Z, et al Activation of proliferator-activated receptors alpha and gamma induces apoptosis of human monocyte-derived macrophages. The Journal of biological chemistry. 1998;273(40):25573–80. 9748221 10.1074/jbc.273.40.25573

[77] Ricote M, Li AC, Willson TM, Kelly CJ, Glass CK. The peroxisome proliferator-activated receptor-gamma is a negative regulator of macrophage activation. Nature. 1998;391(6662):79–82. doi: 10.1038/34178 9422508 10.1038/34178

[78] Staels B, Koenig W, Habib A, Merval R, Lebret M, Torra IP, et al Activation of human aortic smooth-muscle cells is inhibited by PPARalpha but not by PPARgamma activators. Nature. 1998;393(6687):790–3. doi: 10.1038/31701 9655393 10.1038/31701

[79] Zhou YC, Waxman DJ. Cross-talk between janus kinase-signal transducer and activator of transcription (JAK-STAT) and peroxisome proliferator-activated receptor-alpha (PPARalpha) signaling pathways. Growth hormone inhibition of pparalpha transcriptional activity mediated by stat5b. The Journal of biological chemistry. 1999;274(5):2672–81. 9915797 10.1074/jbc.274.5.2672

[80] Jiang C, Ting AT, Seed B. PPAR-gamma agonists inhibit production of monocyte inflammatory cytokines. Nature. 1998;391(6662):82–6. doi: 10.1038/34184 9422509 10.1038/34184

[81] Marx N, Sukhova G, Murphy C, Libby P, Plutzky J. Macrophages in human atheroma contain PPARgamma: differentiation-dependent peroxisomal proliferator-activated receptor gamma(PPARgamma) expression and reduction of MMP-9 activity through PPARgamma activation in mononuclear phagocytes in vitro. The American journal of pathology. 1998;153(1):17–23. PubMed Central PMCID: PMC1852950. 9665460 10.1016/s0002-9440(10)65540-x PMC1852950

[82] Murao K, Imachi H, Momoi A, Sayo Y, Hosokawa H, Sato M, et al Thiazolidinedione inhibits the production of monocyte chemoattractant protein-1 in cytokine-treated human vascular endothelial cells. FEBS letters. 1999;454(1–2):27–30. 10413089 10.1016/s0014-5793(99)00765-6

[83] Momoi A, Murao K, Imachi H, Sayo Y, Nakamura H, Hosokawa H, et al Thiazolidinedione inhibits production of RANTES in a cytokine-treated human lung epithelial cell line. FEBS letters. 1999;452(3):301–4. 10386610 10.1016/s0014-5793(99)00678-x

[84] Jackson SM, Parhami F, Xi XP, Berliner JA, Hsueh WA, Law RE, et al Peroxisome proliferator-activated receptor activators target human endothelial cells to inhibit leukocyte-endothelial cell interaction. Arteriosclerosis, thrombosis, and vascular biology. 1999;19(9):2094–104. 10479650 10.1161/01.atv.19.9.2094

