# Supplementary figures and images for: Is spaceflight-induced immune dysfunction linked to systemic changes in metabolism?
Source: PLoS One. 2017 May 24;12(5):e0174174. doi: 10.1371/journal.pone.0174174 (PMC5443495; doi:10.1371/journal.pone.0174174)

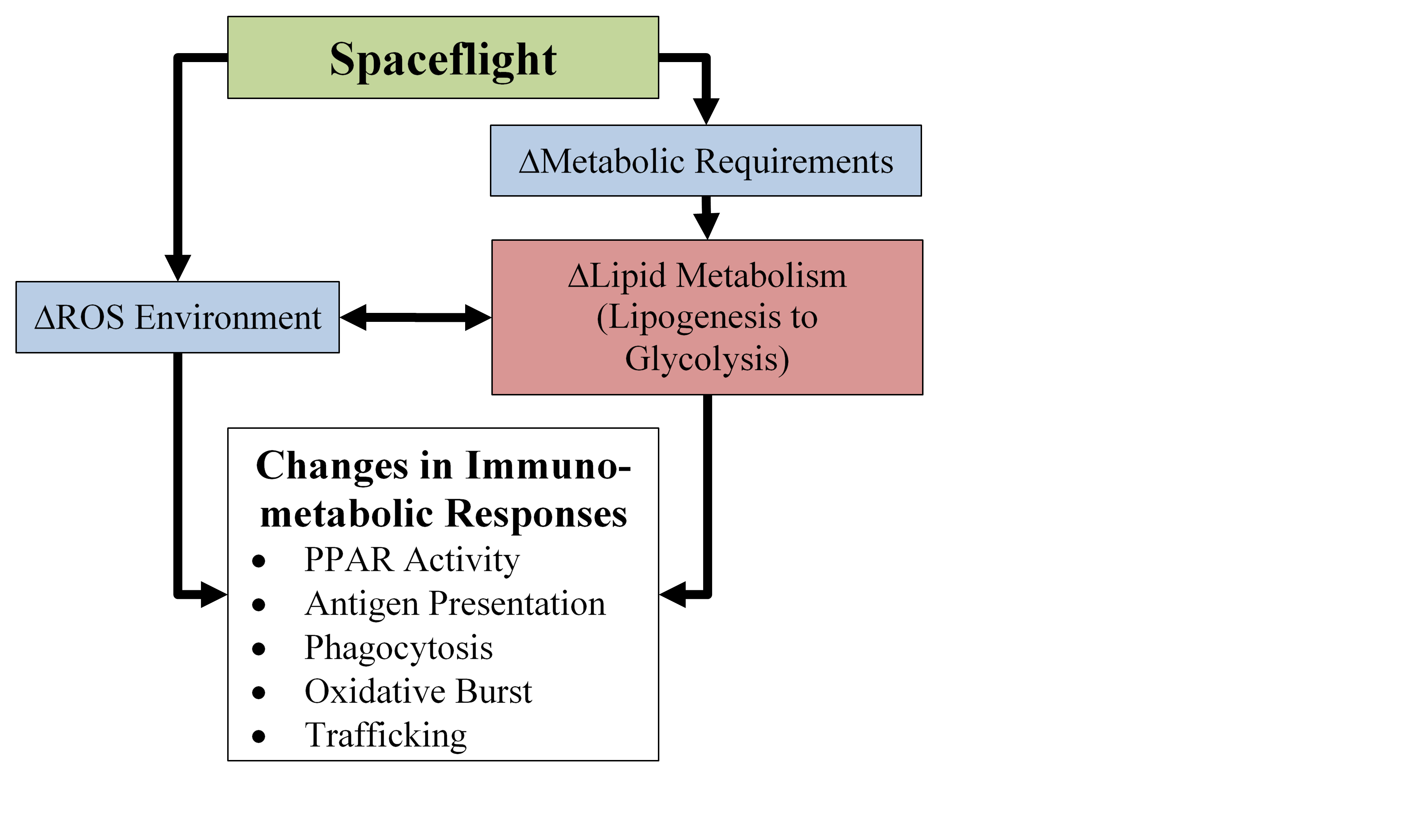

Supplement: S1 Fig — (TIF) [file pone.0174174.s001.tif]

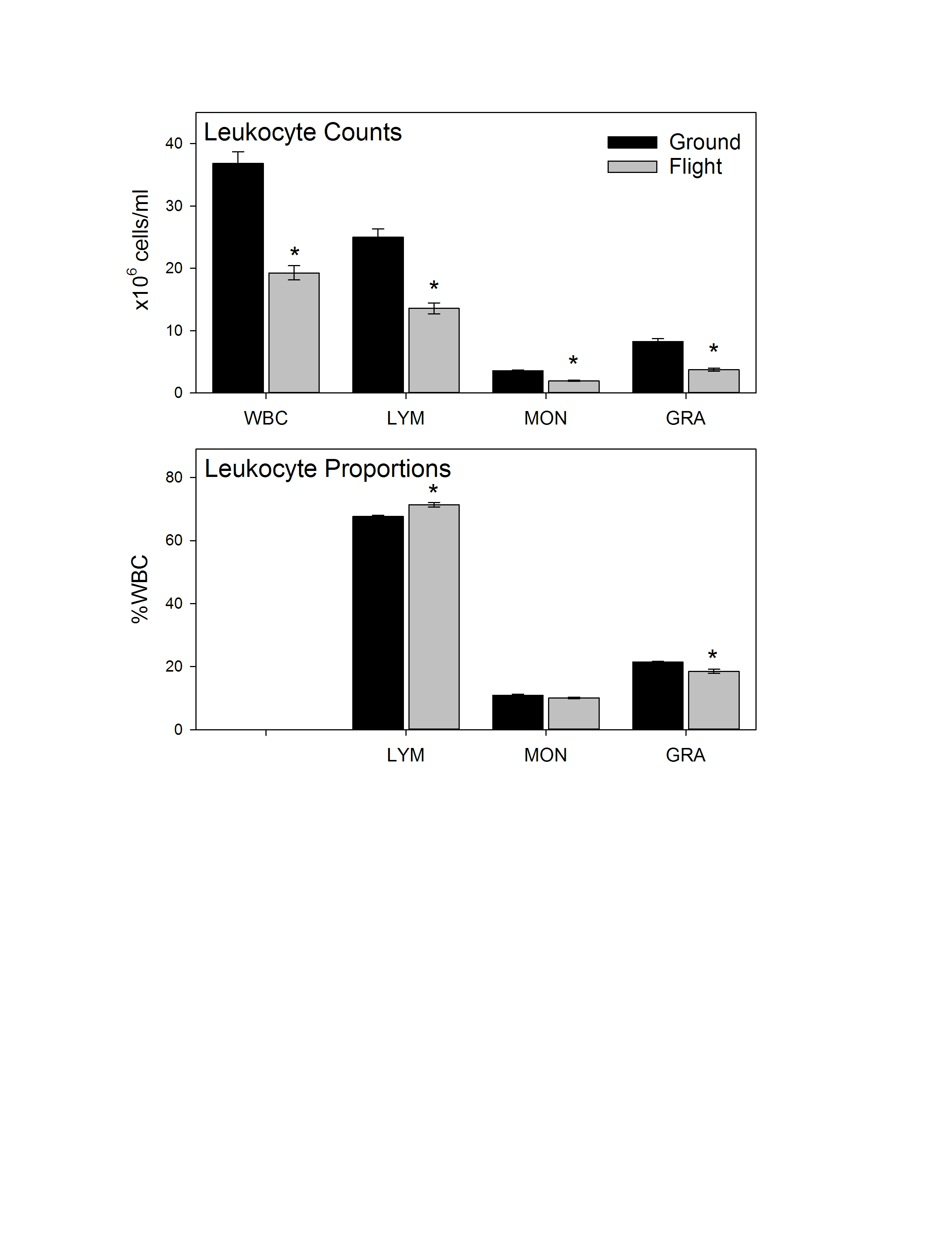

Supplement: S2 Fig — Data were obtained using an automated hematology analyzer. Values were normalized to daily Vivarium controls. WBC = white blood cells. LYM = lymphocytes. MON = monocyte/macrophages. GRA = granulocytes. Values represent means ± SEM. N = 8 for Ground controls housed in animal enclosure modules, 5 for Flight. *P<0.001. (TIF) [file pone.0174174.s002.TIF]

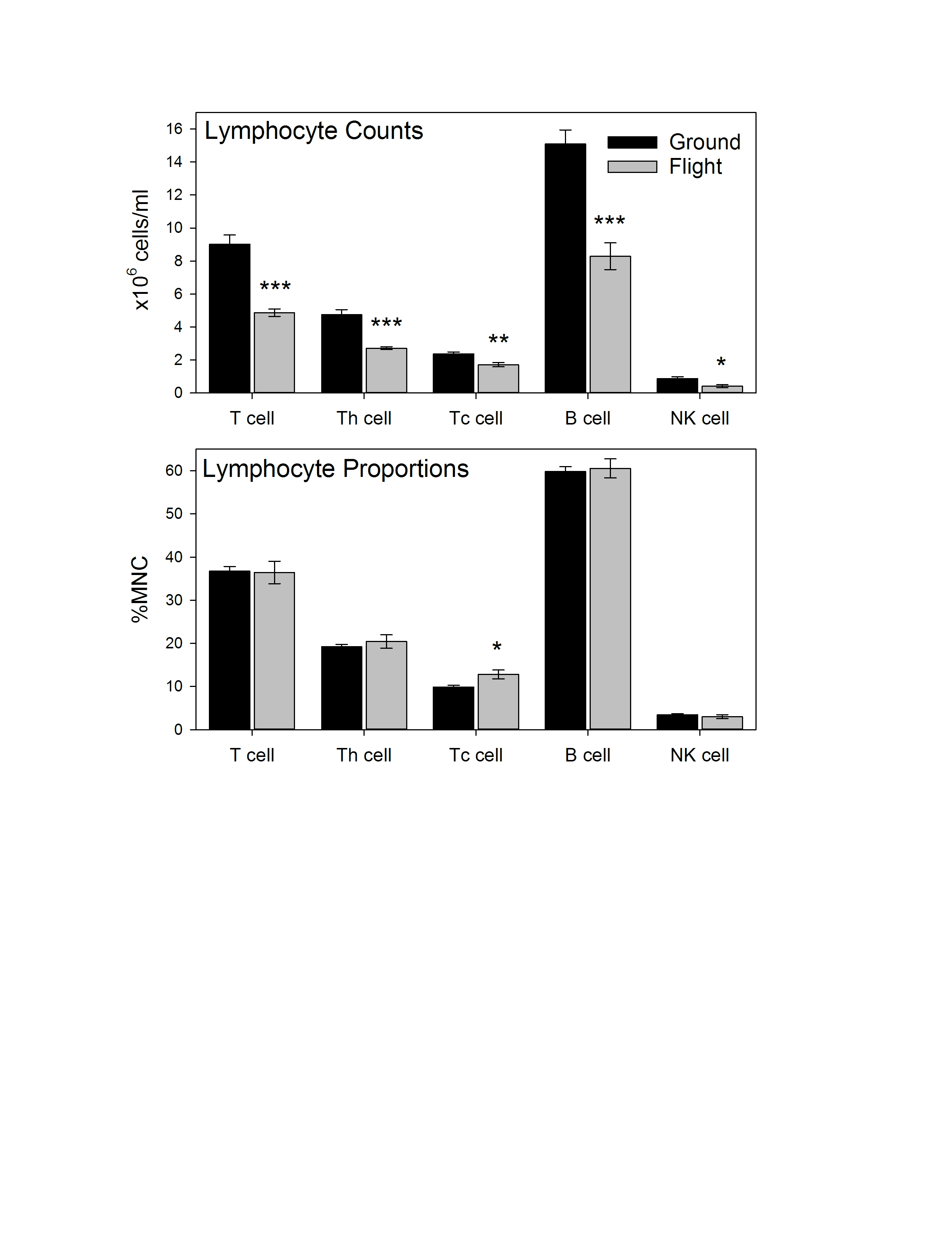

Supplement: S3 Fig — Values represent means ± SEM. N = 8 for Ground controls, 5 for Flight. Values were normalized to daily Vivarium controls. *P<0.05, **P<0.005, ***P<0.001. (TIF) [file pone.0174174.s003.TIF]

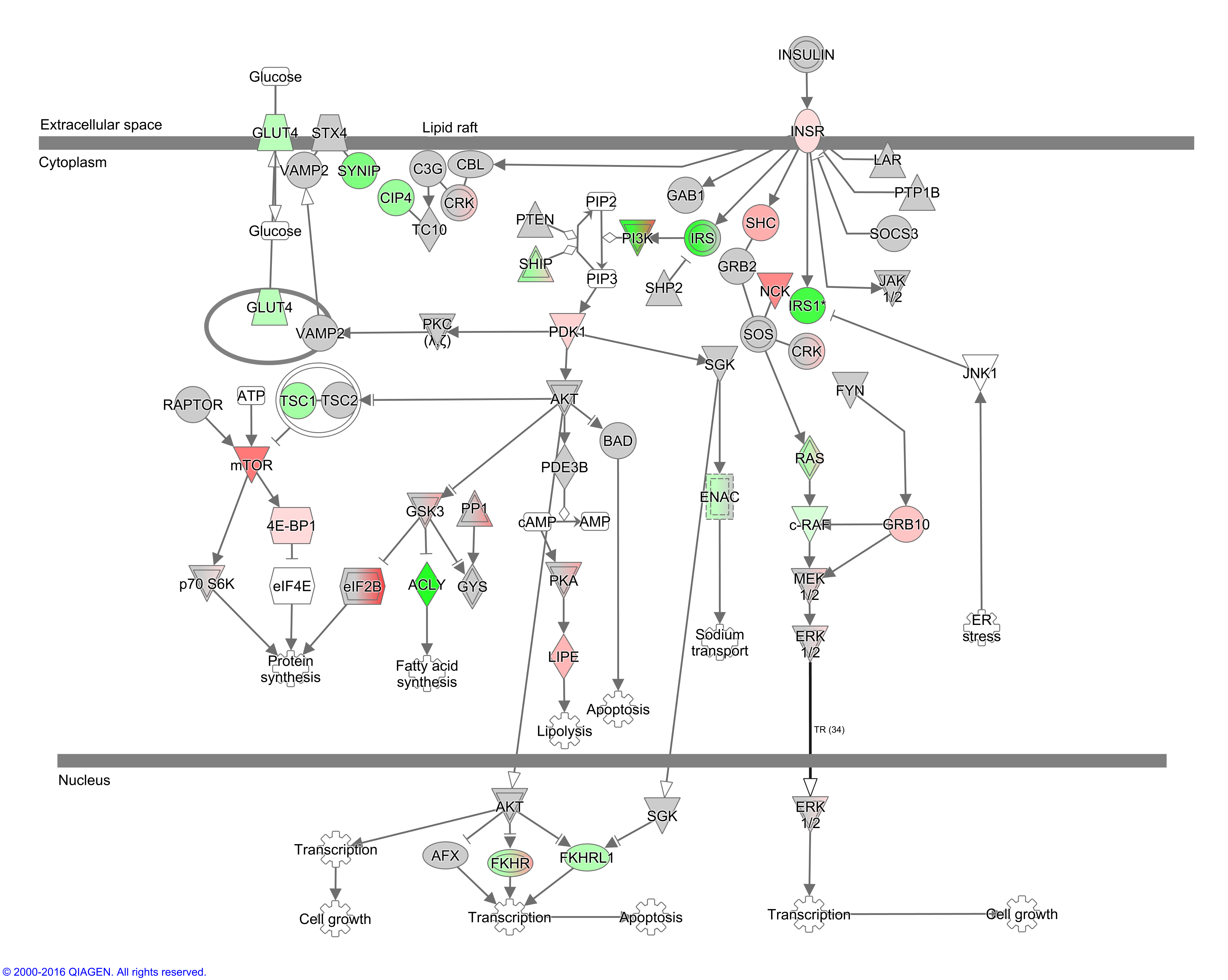

Supplement: S4 Fig — Analysis performed using Ingenuity Pathway Analysis (Qiagen, Inc., Redwood City, CA). Grey = unchanged. Green = down-regulated. Red = up-regulated. (TIF) [file pone.0174174.s004.tif]
