# Supplementary material for: Is spaceflight-induced immune dysfunction linked to systemic changes in metabolism?
Source: PLoS One. 2017 May 24;12(5):e0174174. doi: 10.1371/journal.pone.0174174 (PMC5443495; doi:10.1371/journal.pone.0174174)
Supplement: S1 Table — Values represent means ± SEM. N = 13 for Ground controls, 7 for Flight. NS = Not significant. (DOCX) [file pone.0174174.s005.docx]

**S1 Table. Effect of spaceflight on hormone ratios.** Values represent means ± SEM. N= 13 for Ground controls, 7 for Flight. NS = Not significant.

| **Hormone Ratio** | **Ground** |  | **Flight** |  | **P-Value** |
| --- | --- | --- | --- | --- | --- |
| Corticosterone / ACTH receptor | 4.47 ± 0.68 |  | 6.51 ± 1.19 |  | NS |
| Corticosterone / phosphorylated ACTH receptor | 6.88 ± 1.03 |  | 11.79 ± 2.24 |  | <0.05 |
| Norepinephrine / Corticosterone | 15.66 ± 1.30 |  | 11.89 ± 1.02 |  | 0.068 |
| Epinephrine / Corticosterone | 25.21 ± 2.57 |  | 17.71 ± 1.27 |  | 0.056 |
| Dopamine / Corticosterone | 3.00 ± 0.28 |  | 2.85 ± 0.48 |  | NS |
| Dopamine / Norepinephrine (x100) | 1.90 ± 0.06 |  | 2.32 ± 0.21 |  | <0.05 |
| Epinephrine / Norepinephrine | 1.59 ± 0.05 |  | 1.50 ± 0.04 |  | NS |
