# Supplementary material for: Is spaceflight-induced immune dysfunction linked to systemic changes in metabolism?
Source: PLoS One. 2017 May 24;12(5):e0174174. doi: 10.1371/journal.pone.0174174 (PMC5443495; doi:10.1371/journal.pone.0174174)
Supplement: S1 Discussion — We have analyzed splenic immunocyte populations on three separate space shuttle missions with very similar flight profiles. This additional discussion includes historical context as well as comments on repeatability. (DOCX) [file pone.0174174.s006.docx]

**Supplementary figure legends**

**S1 Fig. Proposed model linking spaceflight-induced disruptions in innate immune function and metabolism.**

**S2 Fig. Effects of spaceflight on splenic leukocyte populations.** Data were obtained using an automated hematology analyzer. Values were normalized to daily Vivarium controls. WBC = white blood cells. LYM = lymphocytes. MON = monocyte/macrophages. GRA = granulocytes. Values represent means ± SEM. N= 8 for Ground controls housed in animal enclosure modules, 5 for Flight. **P<*0.001.

Spaceflight caused decreases in the total number of all major splenic leukocyte population counts. Due to the logistics associated with the tissue sharing after our previous flight, STS-108, we did not have the opportunity to accurately assess splenocyte counts after landing (12 days, *CBTM-1,* 2001) [1]. However, the decreases noted here after STS-135 are similar to our results from STS-118 (*CBTM-2,* 2007) that utilized a mouse model and flight profile nearly identical to the present study [2]. We also found a significant decrease in total leukocyte counts in the spleens isolated from rats after STS-77 (10 days, *Immune-3,* 1996), but there was no significant effect on macrophage counts [3]. Still others have reported spaceflight-induced decreases in total splenocyte counts after STS-57 (10 days, 1993) [4]. Interestingly, the decreases in flight leukocyte counts reported here occurred despite the higher splenic cell viability noted in the flight mice, suggesting that the impact of the shipping error on this response was minimal.

The spleen data are generally consistent with previous flights characterizing these populations in the blood. Chapes, *et al.* reported similar decreases in the count of all three major blood leukocyte subsets in rats after STS-63 (8 days, *Immune-2,* 1995) [5]. Decreases were also reported in blood WBC, monocyte and lymphocyte counts in rats after both STS-58 (14 days, *Spacelab Life Sciences-2 (SLS-2),* 1993) [6] and STS-77 (10 days, *Immune-3*, 1996) [7]. However, neutrophil counts actually increased in both of these flights [6, 7]. Furthermore, except for a decrease in monocyte counts, we found no significant differences in any other major leukocyte count after STS-108 [1]. Similarly, investigators reported no significant difference in circulating leukocyte subset counts after STS-60 (8 days, *Immune-1*, 1994) [5].

Although we did not characterize leukocyte populations in blood from our STS-135 mice, we believe that the spleen may be less susceptible to such acute volatility in population distributions. The differences in the blood data seen across flights is likely due to an acute release of neutrophils from reservoirs (e.g. bone marrow) into the blood shortly after landing. Indeed, this idea is generally consistent with human data [8, 9]. In a recent study characterizing blood leukocyte populations in astronauts 3 hours after landing, sharp increases in total WBC counts were observed that appeared to be driven entirely by an upward shift in granulocytes. As this increase was not found in blood samples taken 24 hours before landing, the change was likely an acute response to the landing [9].

When viewed as percentages, there was a slight shift in splenocytes away from granulocytes and toward lymphocytes. Although this is consistent with our results from STS-108 [1], we did not see this shift after STS-118 [2]. The lack of a shift in the spleen after STS-118 may also be due to some of the post-flight handling. Unlike STS-108 and -135, the mice on STS-118 were evaluated for muscle strength and underwent nuclear magnetic resonance (NMR) imaging as part of the primary science protocol. The muscle test (described here [10]) involved a brief restraint and the use of a small electric stimulus. This additional handling prior to euthanasia may have caused an acute stress response significant enough to alter splenic leukocyte trafficking.

While there are reports of similar shifts in the blood leukocytes toward lymphocytes and away from neutrophils [5, 11], the reverse response has also been reported in both rodents [6, 7] and humans [8, 12]. As discussed above, the variability of responses in the blood noted in some previous flights is likely due to differences in post-landing stress.

**S3 Fig. Effects of spaceflight on lymphocyte subsets.** Values represent means ± SEM. N= 8 for Ground controls, 5 for Flight. Values were normalized to daily Vivarium controls. **P<*0.05, ***P<*0.005, ****P<*0.001.

Spaceflight caused decreases in all splenic T and B cell counts. These decreases in cell number are nearly identical to our previous results from STS-118 that utilized an animal model and flight profile very similar to the present study [13]. In contrast, we did not find any significant effect of spaceflight on T, Th or Tc counts in rats after STS-77 [3]. Although Ichiki *et al*. did not report splenocyte counts (only percentages in the spleen were reported), they found decreases in CD3+ T, CD4+ Th, CD8+ Tc and B cell counts in the blood after STS-58 [6].

There was no significant impact of spaceflight on the proportions of splenic T or B cells. There was a similar lack of a response after STS-58 in the T and B subsets [6]. This contrasts our results from STS-77 and -118, where we saw decreases in splenic T cell percentages [3, 13], and STS-108, where we found a slight shift away from T cells toward B cells [1]. In contrast, while there were increases in the spleen for both T (CD4+ and CD8+) and B (kappa+) cell proportions in rats after STS-57, the increases appear to be greater in the T cell subsets [4]. However, similar to our findings in the present study, there were no significant changes in these populations in the lymph nodes of these very same animals [4]. Interestingly, Crucian *et al*. also reported an increase in B cell percentages in astronaut blood after flight [12]. Finally, unlike the present flight, we saw increases in NK cell proportions after both STS-108 and -118 [1, 13].

Within the T cells, there was a slight increase in Tc proportions that led to a decrease in the CD4/CD8 ratio. We found similar decreases in the ratio after both STS-77 [3] and -108 [1]. However, after STS-108, the decrease was due to decreases in Th cell proportions rather than increases in Tc cells [1]. Although we found no significant difference in the ratio after STS-118 [13], this lack of a response was likely due to additional handling stress in this flight discussed previously.

It should be noted that while the CD4/CD8 ratio was not directly reported in previous studies, calculating the ratio from the presented data suggests that the ratio increased after flight in the blood of astronauts [12] and rats [6], and either increased [4] or remained unchanged [6] in the spleens of rats. Because these are percentages, the slight discrepancies in individual population proportions between flights may simply be due to differences in antibodies or the placement of the gates during flow cytometric analysis. Another possible explanation is the animal model. Most rodent flights prior to STS-108 involved rats whereas our studies on STS-108, -118, and -135 all involved mice.

**S4 Fig. Impact of spaceflight on the insulin signaling pathway.** Analysis performed using Ingenuity Pathway Analysis (Qiagen, Inc., Redwood City, CA). Grey = unchanged. Green = down-regulated. Red = up-regulated.

**References for supplemental data section**

1. Pecaut MJ, Nelson GA, Peters LL, Kostenuik PJ, Bateman TA, Morony S, Stodieck LS, Lacey DL, Simske SJ, and Gridley DS, *Genetic Models in Applied Physiology: Selected Contribution: Effects of spaceflight on immunity in the C57BL/6 mouse. I. Immune population distributions.* J Appl Physiol, 2003. **94**(5): p. 2085-2094.

2. Baqai FP, Gridley DS, Slater JM, Luo-Owen X, Stodieck LS, Ferguson V, Chapes SK, and Pecaut MJ, *Effects of spaceflight on innate immune function and antioxidant gene expression.* J Appl Physiol (1985), 2009. **106**(6): p. 1935-42.

3. Pecaut MJ, Simske SJ, and Fleshner M, *Spaceflight induces changes in splenocyte subpopulations: effectiveness of ground-based models.* Am J Physiol Regulatory Integrative Comp Physiol, 2000. **279**(6): p. R2072-R2078.

4. Grove DS, Pishak SA, and Mastro AM, *The effect of a 10-day space flight on the function, phenotype, and adhesion molecule expression of splenocytes and lymph node lymphocytes.* Exp Cell Res, 1995. **219**(1): p. 102-9.

5. Chapes SK, Simske SJ, Sonnenfeld G, Miller ES, and Zimmerman RJ, *Effects of space flight and PEG-IL-2 on rat physiological and immunological responses.* J Appl Physiol, 1999. **86**(6): p. 2065-2076.

6. Ichiki AT, Gibson LA, Jago TL, Strickland KM, Johnson DL, Lange RD, and Allebban Z, *Effects of spaceflight on rat peripheral blood leukocytes and bone marrow progenitor cells.* J Leukoc Biol, 1996. **60**(1): p. 37-43.

7. Chapes SK, Simske SJ, Forsman AD, Bateman TA, and Zimmerman RJ, *Effects of space flight and IGF-1 on immune function.* Adv Space Res, 1999. **23**(12): p. 1955-1964.

8. Stowe RP, Sams CF, Mehta SK, Kaur I, Jones ML, Feeback DL, and Pierson DL, *Leukocyte subsets and neutrophil function after short-term spaceflight.* J Leukoc Biol, 1999. **65**(2): p. 179-86.

9. Crucian B, Stowe R, Mehta S, Uchakin P, Quiriarte H, Pierson D, and Sams C, *Immune system dysregulation occurs during short duration spaceflight on board the space shuttle.* J Clin Immunol, 2013. **33**(2): p. 456-65.

10. Stodieck LS, Greybeck BJ, Cannon CM, Hanson AM, Young MH, Simske SJ, and Ferguson VL, *In vivo measurement of hindlimb neuromuscular function in mice.* Muscle Nerve, 2012. **45**(4): p. 536-43.

11. Lange RD, Andrews RB, Gibson LA, Congdon CC, Wright P, Dunn CD, and Jones JB, *Hematological measurements in rats flown on Spacelab shuttle, SL-3.* Am J Physiol, 1987. **252**(2 Pt 2): p. R216-21.

12. Crucian BE, Stowe RP, Pierson DL, and Sams CF, *Immune system dysregulation following short- vs long-duration spaceflight.* Aviat Space Environ Med, 2008. **79**(9): p. 835-43.

13. Gridley DS, Slater JM, Luo-Owen X, Rizvi A, Chapes SK, Stodieck LS, Ferguson VL, and Pecaut MJ, *Spaceflight effects on T lymphocyte distribution, function and gene expression.* J Appl Physiol, 2009. **106**(1): p. 194-202.
